# Global satellite analysis reveals landslide susceptibility peaks at intermediate vegetation density

**DOI:** 10.1016/j.isci.2026.117271

**Published:** 2026-08-20

**Authors:** Shaoxiong Yuan, Quan Wang, Qinghua Gong, Miguel A. Gonzalez-Rodriguez, Guangman Song, Yongqiang Zong, Yinlei Hao, Jun Wang, Yu Chen

**Affiliations:** 1 Guangzhou Institute of Geography/Guangdong Emergency Technology Research Center for Geological Disasters, Guangdong Academy of Sciences, Guangzhou 510070, China; 2 Faculty of Agriculture, Shizuoka University, Shizuoka 422-8529, Japan; 3 School of Architecture and Urban Planning, Guangdong University of Technology, Guangzhou 510062, China; 4 College of Ecology and Environment, Nanjing Forestry University, Nanjing 210037, China

**Keywords:** landslide susceptibility, NDVI, vegetation paradox, critical transition zone, atellite remote sensing, slope stability, breakpoint analysis

## Abstract

Landslide hazard models worldwide assume that vegetation stabilizes slopes monotonically, so that denser vegetation always implies greater stability. Analyzing 12,459 rainfall-triggered landslides (2000–2024) with multi-source satellite data, we find a “vegetation paradox”: landslide susceptibility does not decline monotonically with vegetation density but peaks within a critical transition zone (CTZ) at NDVI, 0.769–0.868—a unimodal, non-monotonic pattern. This zone concentrates 23.0% of the full 18,028-event inventory (33.2% of the 12,459 analyzed with reliable NDVI) and persists across biomes, climate zones, and slope classes (16.7%–44.5% CTZ prevalence across the gentle-to-steep range), and its upper boundary is stable across data-quality filtering, density smoothing, and simulated sensor noise. An independent grid-based Random Forest (*R*^2^ = 0.446) independently places this maximum within the CTZ (mean NDVI, 0.860) without histogram binning; its coarse grid, however, captures only the rising limb and cannot resolve the decline above the CTZ.

## Highlights

• Landslide risk peaks at intermediate vegetation, not in barren terrain
• A critical transition zone at NDVI, 0.769–0.868 concentrates 23% of events
• Independent Random Forest validation confirms the non-monotonic pattern
• The vegetation paradox persists across biomes, climates, and slopes

## Graphical abstract

**Figure:**
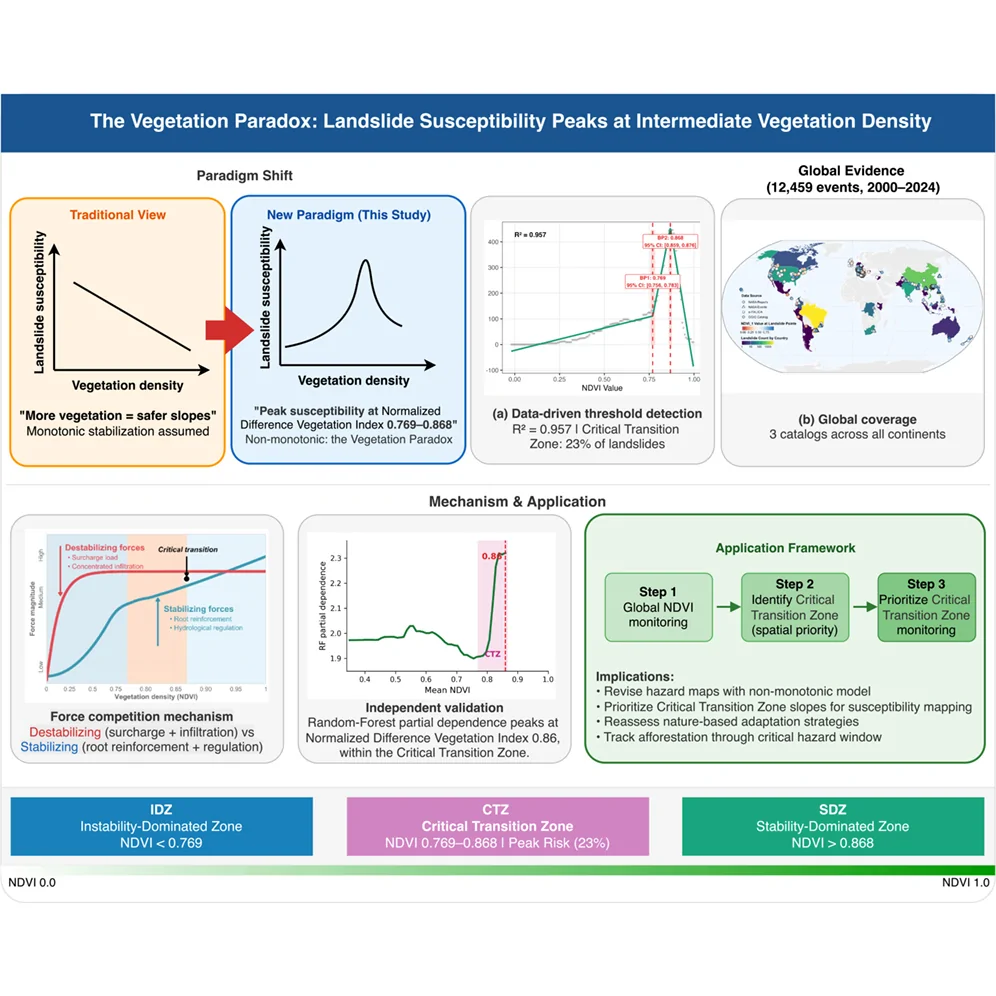

## Teaser

Environmental science; Environmental monitoring

## Introduction

Landslides represent one of the most destructive natural hazards globally, killing more than 4,000 people annually^1^^,^^2^ and contributing to the escalating economic toll of climate-related extreme-weather events.^3^ Recent analyses indicate that climate change will intensify landslide risks through altered precipitation patterns, with projected 10%–25% increases in landslide frequency across vulnerable regions by 2050.^1^^,^^4^^,^^5^ The IPCC Sixth Assessment Report specifically identifies landslide hazards as a critical climate adaptation challenge, particularly in tropical and subtropical mountainous regions where population growth and infrastructure development continue to expand.^6^^,^^7^^,^^8^ Against this backdrop, developing robust, physically informed predictive frameworks for landslide susceptibility assessment has emerged as an urgent scientific and societal imperative.^9^^,^^10^

Vegetation’s role in slope stability has traditionally been conceptualized through a dual-mechanism framework established over four decades of geotechnical research.^11^^,^^12^^,^^13^^,^^14^ Mechanical stabilization occurs through root reinforcement, where fibrous root networks increase soil cohesion by 15%–150%, depending on species and root architecture.^15^^,^^16^^,^^17^ Hydrological stabilization involves canopy interception (reducing soil water input by 10%–40%), root water uptake (lowering pore water pressure), and enhanced evapotranspiration.^18^^,^^19^^,^^20^^,^^21^ This mechanistic understanding has crystallized into a foundational assumption underlying most landslide susceptibility models: that vegetation density, commonly quantified using satellite-derived indices such as the normalized difference vegetation index (NDVI), exhibits a monotonically inverse relationship with landslide probability^22^^,^^23^^,^^24^^,^^25^^,^^26^—a foundational yet largely untested assumption in global-scale modeling. This assumption underpins both statistical^27^^,^^28^ and physically based^29^^,^^30^ modeling approaches worldwide.

However, decades of field observations have documented complex vegetation-landslide interactions that challenge this monotonic assumption.^31^^,^^32^^,^^33^ Individual studies have identified destabilizing effects of certain vegetation characteristics, including biomass surcharge loading on slopes,^34^^,^^35^ concentrated throughfall and stemflow that can trigger rapid soil saturation,^36^^,^^37^ and root-induced preferential flow paths that may accelerate subsurface water movement.^38^^,^^39^ More recently, satellite-based studies have identified anomalous pre-failure vegetation dynamics—including both stress-induced browning and counterintuitive greening—that may signal heightened landslide susceptibility.^40^^,^^41^^,^^42^ These isolated observations suggest that vegetation-landslide relationships may exhibit critical thresholds delineating stabilizing versus destabilizing regimes,^43^^,^^44^ yet the broader implications of these findings have remained largely unexplored at the global scale.

While these site-specific studies have provided valuable mechanistic insights, systematic global-scale quantification of vegetation-landslide relationships using objective, data-driven threshold detection methods remains absent from the literature.^2^^,^^45^ The transition from isolated field observations to universally applicable predictive frameworks has been hindered by fundamental methodological limitations: previous studies have been limited by small sample sizes,^46^^,^^47^ regional focus,^48^^,^^49^ or subjective threshold selection methods.^50^^,^^51^ Most critically, no study has employed statistically robust, objective algorithms to identify critical vegetation density breakpoints using comprehensive global datasets. This methodological gap represents a fundamental constraint on our ability to incorporate vegetation dynamics into next-generation landslide prediction systems,^52^^,^^53^ leaving the slope stability community without validated thresholds for operational hazard assessment.

Here, we address this fundamental limitation through what is, to our knowledge, the first global-scale, data-driven analysis of vegetation-landslide relationships using objective threshold detection methods. We have assembled a database of 18,028 rainfall-triggered landslide events (2000–2024; 12,459 with reliable pre-failure NDVI) integrated with multi-temporal satellite observations from MODIS, Sentinel-2, and complementary Earth observation platforms. Our approach employs segmented regression techniques to objectively identify critical vegetation density thresholds, validates findings across multiple land cover products and climate zones, and develops a predictive modeling framework to independently confirm discovered relationships. This analysis reveals a unimodal, non-monotonic vegetation-landslide relationship—the “vegetation paradox”—that challenges traditional assumptions and provides validated tools for enhanced global landslide hazard assessment.

## Results

### Data-driven discovery of a unimodal vegetation-landslide relationship

**Figure 1:**
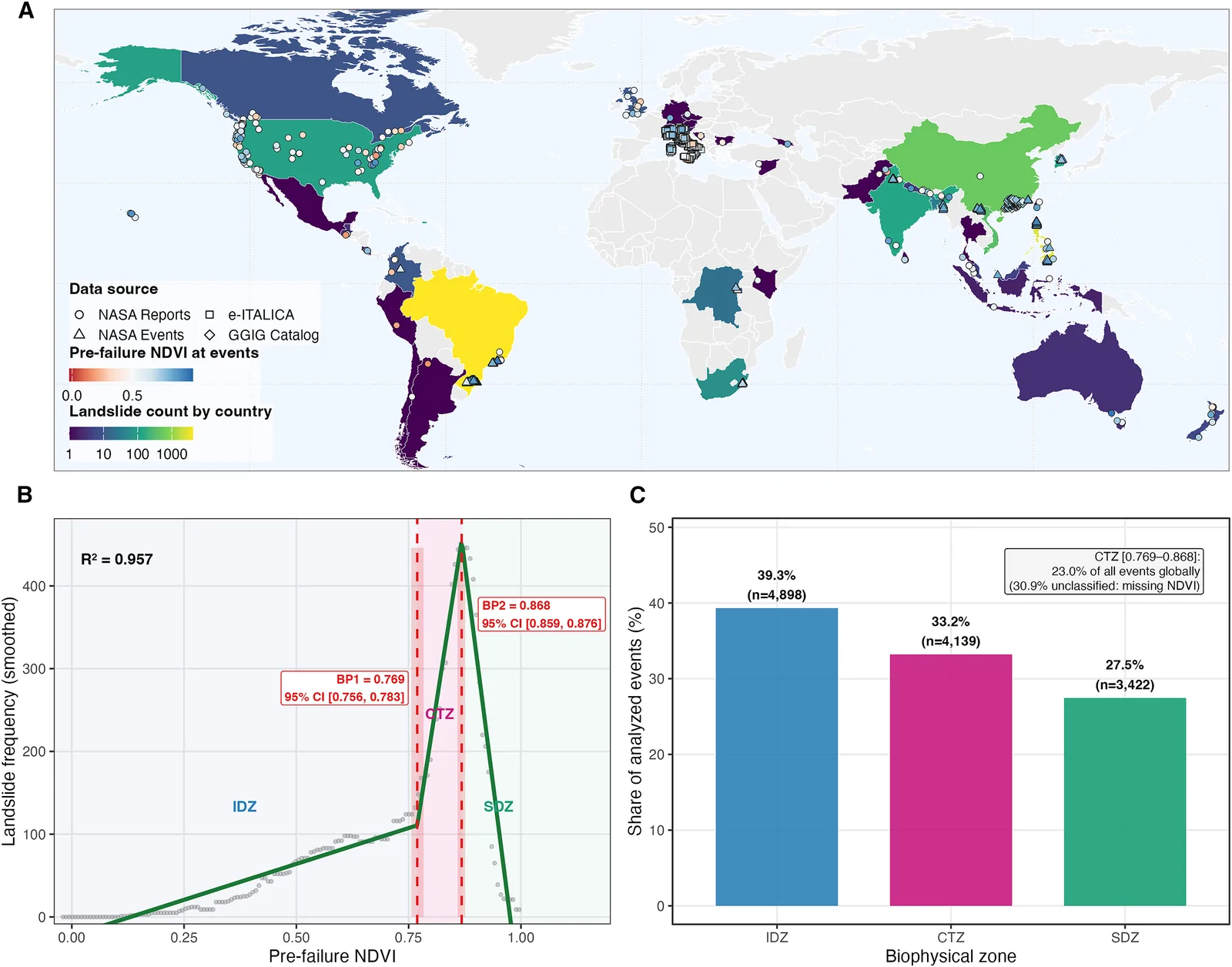
Discovery of a unimodal, non-monotonic vegetation-landslide relationship (A) Global distribution of the rainfall-triggered landslide inventory (2000–2024); each event is colored by its pre-failure NDVI and symbol-coded by data source, and country shading gives the per-country event count. (B) Smoothed pre-failure NDVI frequency distribution for the 12,459 events with reliable NDVI, with a two-breakpoint segmented fit (*R*^2^ = 0.957, *p* < 0.001). The breakpoints BP1 = 0.769 (95% bias-corrected and accelerated (BCa) bootstrap CI 0.756–0.783) and BP2 = 0.868 (0.859–0.876) define three zones—the instability-dominated zone (IDZ), critical transition zone (CTZ), and stability-dominated zone (SDZ); frequency rises to a peak within the CTZ before declining (red bands, 95% CIs). (C) Share of events per zone: IDZ, 39.3%; CTZ, 33.2%; and SDZ, 27.5% (equivalently 27.2%, 23.0%, and 19.0% of the full 18,028-event inventory; the remaining 30.9% lacked reliable NDVI). Enhanced vegetation index (EVI) and leaf area index (LAI) analyses appear in Figure S2.

We compiled 18,028 rainfall-triggered landslide events spanning 2000–2024 and extracted pre-failure NDVI values for each from MODIS 16-day composites (STAR Methods; Figure 1A); 12,459 events (69.1%) had reliable NDVI and formed the core dataset. Along the vegetation gradient, landslide frequency followed a pronounced unimodal distribution: it rose with NDVI to a distinct peak at moderate-to-high vegetation density and then declined, contrary to the long-assumed monotonic decrease (Figure 1B).

Segmented regression of the smoothed frequency distribution (STAR Methods; Figure 1B) identified two statistically significant breakpoints, BP1 = 0.769 (95% confidence interval [CI], 0.756–0.783) and BP2 = 0.868 (0.859–0.876), with *R*^2^ = 0.957 (adjusted *R*^2^ = 0.955; residual standard error 23.5 on 144 degrees of freedom; *p* < 0.001; Figure S1; Table S1). This *R*^2^ = 0.957—the fit of the two-breakpoint model to the smoothed frequency distribution—is the authoritative value reported consistently across the text, figures, and tables. These breakpoints define three biophysical zones (Figure 1C). In the instability-dominated zone (IDZ; NDVI <0.769), landslide frequency is high and *increases* with vegetation density, encompassing 39.3% of analyzed events (27.2% of the full inventory). The critical transition zone (CTZ; NDVI, 0.769–0.868) spans only 0.099 in NDVI yet concentrates 33.2% of analyzed events (23.0% of the full inventory)—a disproportionate density that constitutes the core of the “vegetation paradox,” in which the maximum of landslide frequency occurs not at the lowest vegetation density but at moderate-to-high density. These shares describe how observed failures are distributed along the vegetation gradient and should not be read as a per-unit-area hazard rate, which would additionally require the land area available at each NDVI (see limitations of the study). In the stability-dominated zone (SDZ; NDVI >0.868), frequency then declines sharply, encompassing 27.5% of analyzed events (19.0% of the full inventory); the remaining 30.9% of the inventory lacked reliable NDVI (see limitations of the study).

The same two-breakpoint structure is recovered with EVI and LAI (Figure S2) and corroborated by generalized additive models whose turning points coincide with the segmented breakpoints (Figures S3–S5). Because NDVI and EVI saturate at high canopy density, their breakpoints fall at different index values, but all three indices place the susceptibility maximum at intermediate-to-high vegetation density (Figure S2; Table S2).

### Multi-platform and cross-biome robustness

**Figure 2:**
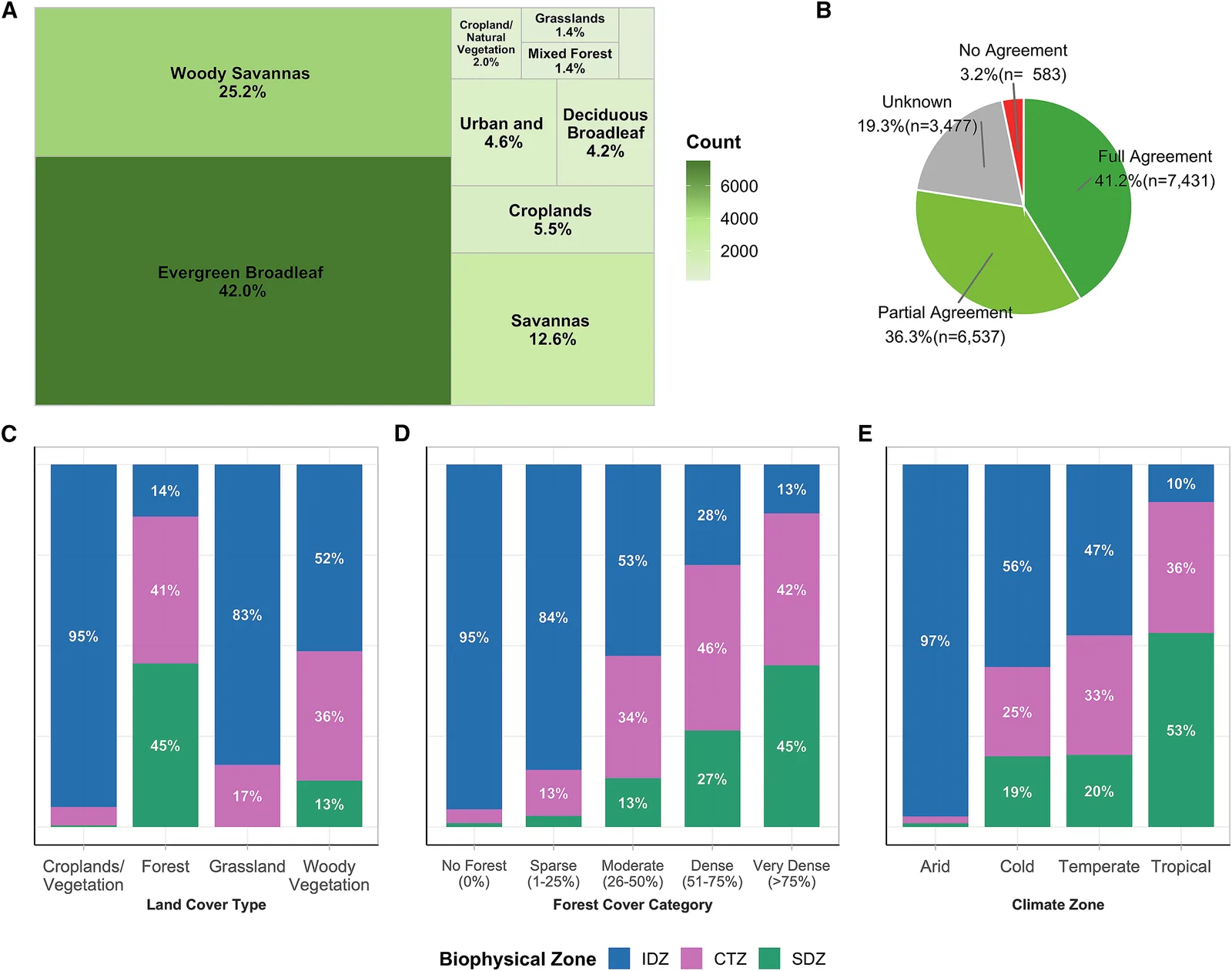
Multi-platform and cross-biome validation (A) Distribution of MODIS IGBP vegetation types at the landslide locations. (B) Cross-platform land-cover agreement among the MODIS, Copernicus, and Hansen classifications. (C–E) The three-zone structure (IDZ/CTZ/SDZ) is reproduced when events are stratified by land-cover type (C), forest-cover density (D), and Köppen climate zone (E), showing the pattern persists across biomes, forest densities, and climate zones.

Landslides in our inventory occur across the full range of vegetated land-cover types (Figure 2A), and the three independent land-cover products—MODIS, Copernicus, and Hansen—classify these locations consistently (Figure 2B; full classification flow in Figure S6), providing a robust basis for testing the pattern across datasets.

The three-zone structure is not tied to any single vegetation dataset or biome. When events are stratified independently by land-cover type (Figure 2C), by forest-cover density (Figure 2D), and by Köppen climate zone (Figure 2E), the elevated CTZ frequency re-emerges under every classification, and the breakpoints are independently recovered within individual climate zones (Figure S7). The unimodal pattern is therefore a general property of the vegetation-landslide system rather than of one sensor, land-cover scheme, or climatic setting.

The breakpoints are equally robust to data quality, smoothing, and sensor noise. They are essentially unchanged when restricted to cloud-free observations (MODIS QA = 0, *n* = 9,168), computed separately by continent, or with high-missingness monsoon-Asia events excluded (BP1, 0.762–0.772; BP2, 0.865–0.876, all within the full-sample bootstrap CI; Figure S8; Table S3); across 12 binning and kernel-bandwidth choices (BP2, 0.866–0.868; BP1, 0.72–0.78; Table S4); and under Monte-Carlo MODIS noise up to *σ* = 0.05, an order of magnitude above the nominal MODIS uncertainty (BP2 shift <0.003; the more sensitive BP1 ≤0.03 at realistic noise; Figure S9; Table S5). The CTZ width (0.099) thus exceeds the combined boundary uncertainty.

### Vegetation, climate, and seasonal structure of landslide occurrence

**Figure 3:**
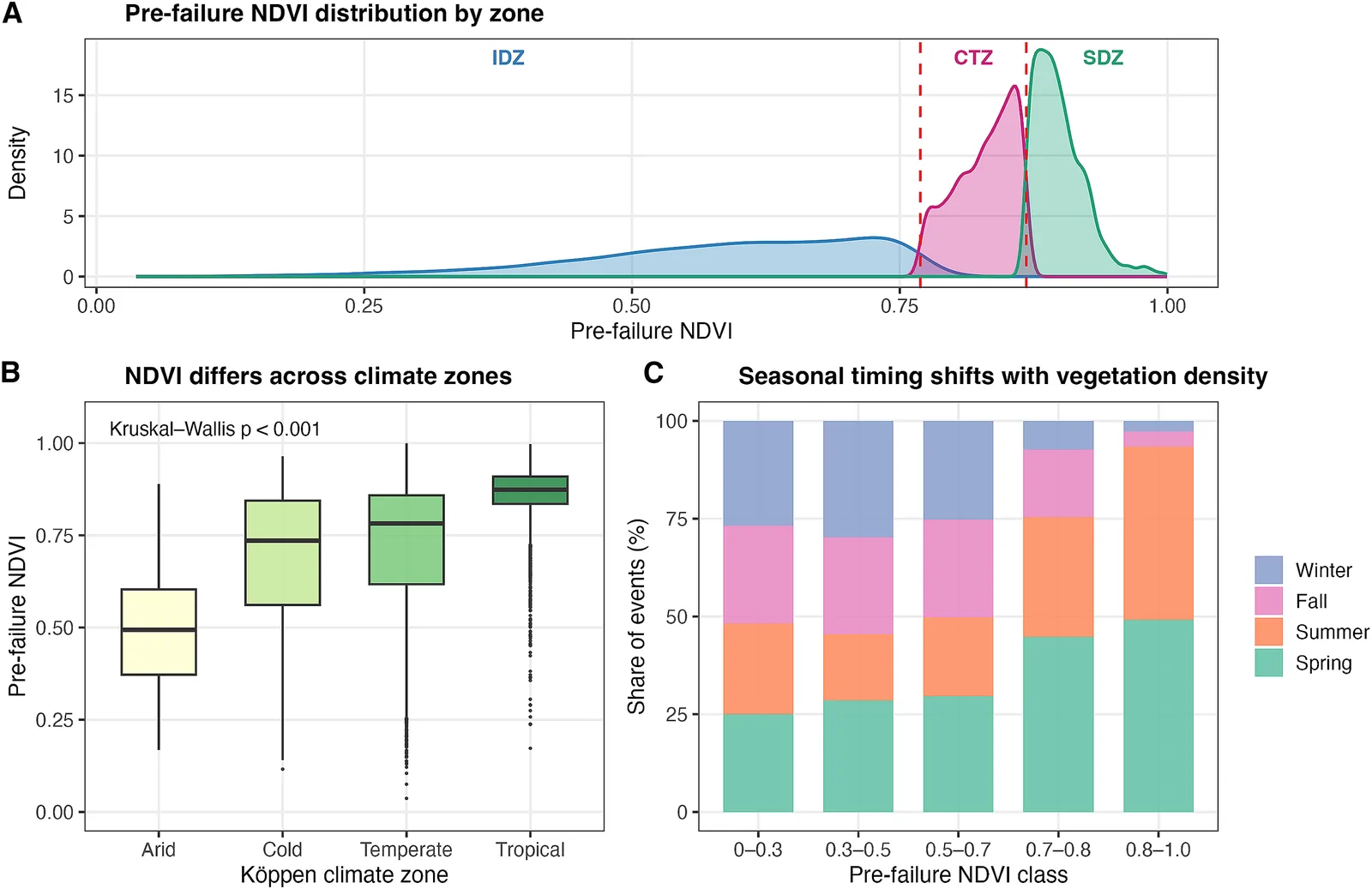
Vegetation, climate, and seasonal structure of landslide occurrence (A) Pre-failure NDVI density distributions for each biophysical zone (dashed lines: breakpoints BP1 = 0.769, BP2 = 0.868). (B) Pre-failure NDVI differs significantly across Köppen climate zones (Kruskal-Wallis rank-sum test, *p* < 0.001; *n* = 12,459 events). Boxplots show the median (center line), the interquartile range (box), whiskers extending to at most 1.5× the interquartile range, and individual points beyond that range. (C) Seasonal timing of failure shifts from near-uniform at low NDVI to strongly seasonal at high NDVI, where failures concentrate in spring and summer—reflecting coupled rainfall and vegetation phenology in monsoon-influenced regions. A separate test for a pre-failure NDVI precursor, and its sensitivity to post-failure compositing contamination, is reported in Figure S10 and Table S6 (see results).

The three biophysical zones separate cleanly in pre-failure NDVI (Figure 3A), and pre-failure NDVI varies significantly across Köppen climate zones (Kruskal-Wallis, *p* < 0.001; Figure 3B), reflecting the different baseline vegetation densities of the biomes in which rainfall-triggered failures occur. Failure timing is likewise coupled to vegetation density (Figure 3C): in low-NDVI environments (0–0.3) failures are nearly aseasonal (*χ*^2^ = 0.4, *p* = 0.93), whereas in high-NDVI environments (0.8–1.0) they are strongly seasonal (*χ*^2^ = 5,155.6, *p* < 0.001), concentrated in spring (49%) and summer (44%). Because high-NDVI events cluster in monsoon regions, this timing reflects coupled rainfall and vegetation phenology (see limitations of the study).

We also examined whether NDVI declines measurably before failure. Stratifying each event’s terminal (0–16 days) NDVI window by zone produces an apparent, zone-ordered decline (−0.029, −0.018, and +0.018 NDVI units for the IDZ, CTZ, and SDZ; Tables S6 and S7), but this is an artifact of the MODIS compositing window, which straddles the failure date and captures the fresh, bare-soil landslide scar for 96% of events (STAR Methods; Figure S10A). Restricted to windows lying entirely before failure (≥16 days prior), the decline disappears (terminal step +0.002, +0.008, and +0.003; the within-event anomaly is negligible in every zone—≤0.003 NDVI units, non-significant in the IDZ (*p* = 0.19) and CTZ (*p* = 0.34), and marginally significant but of trivial magnitude in the SDZ (*p* = 0.03); Figure S10B; Table S6), and the breakpoint structure is unchanged across all windows (BP1, 0.74–0.81; BP2, 0.87–0.90). We therefore do not interpret the terminal decline as an early-warning signal.

## Discussion

### The vegetation paradox and its geotechnical basis

**Figure 4:**
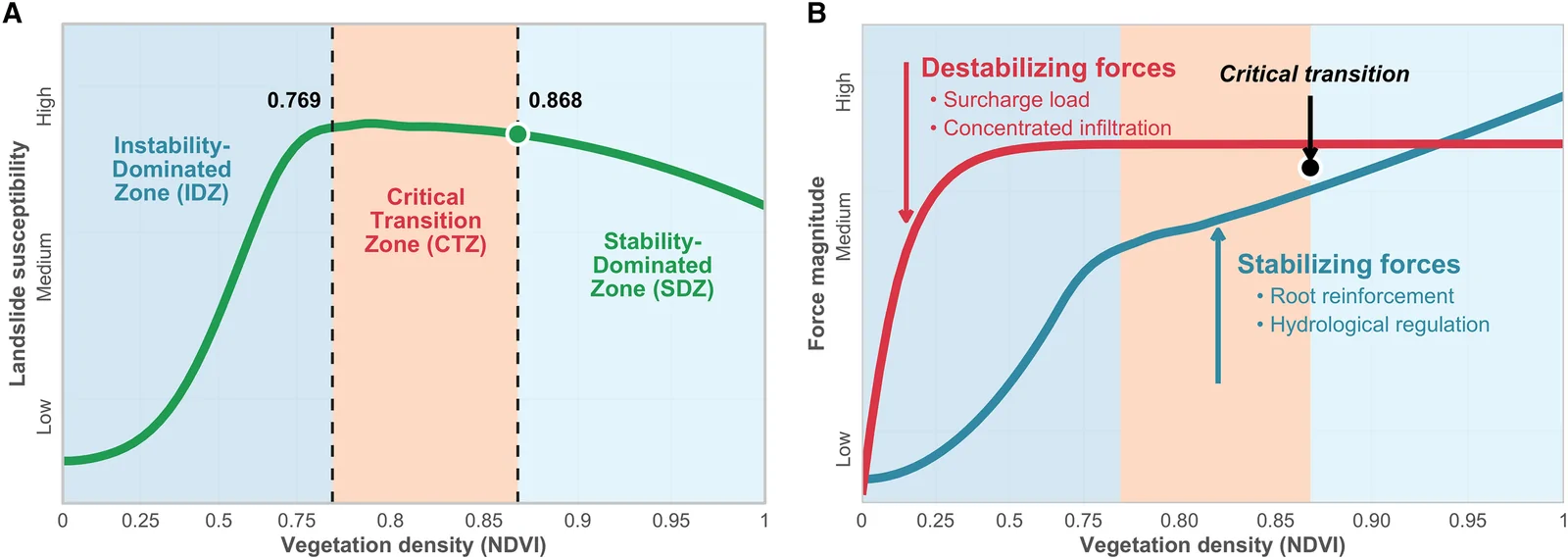
The vegetation paradox framework (A) The non-monotonic (unimodal) susceptibility curve: landslide susceptibility rises to a peak within the critical transition zone (CTZ; NDVI, 0.769–0.868) before declining. (B) Proposed competition between stabilizing forces (root reinforcement and hydrological regulation) and destabilizing forces (biomass surcharge load and concentrated infiltration), whose crossover within the CTZ density band offers a conceptual account of the paradox. This framework is a hypothesis for the spatial (cross-sectional) susceptibility pattern; we make no temporal early-warning claim (see results and Figure S10).

Our global analysis establishes statistically validated, objectively derived vegetation density thresholds for landslide susceptibility assessment. Through the analysis of 12,459 rainfall-triggered events, we demonstrate that the vegetation-landslide relationship is fundamentally non-monotonic. This is characterized by the identification of a CTZ at NDVI values between 0.769 and 0.868. We propose the vegetation paradox framework (Figure 4), which conceptualizes this relationship as a competition between stabilizing and destabilizing forces.

The identification of the IDZ (NDVI <0.769) as a high-susceptibility zone resolves an apparent contradiction in the literature.^11^^,^^12^^,^^13^^,^^14^ Foundational geotechnical studies were predominantly conducted in moderately to densely vegetated environments where root reinforcement effects dominate.^15^^,^^16^^,^^17^ Our global analysis reveals that in sparsely vegetated terrains, vegetation density falls below the critical threshold needed for effective slope stabilization^43^^,^^44^; consistent with this, InSAR-based inventories in semi-arid terrain find landslide deformation concentrated in sparsely vegetated areas (NDVI <0.3).^54^

### A force-balance hypothesis for the CTZ

Theoretically, the CTZ concept builds upon established soil bioengineering principles^13^^,^^14^ and ecohydrological slope stability theory.^20^^,^^21^ Within this zone, we hypothesize a critical competition between progressive stabilizing forces—increasing root density and tensile strength,^55^^,^^56^ enhanced soil-root matrix cohesion^15^^,^^16^—and concurrent destabilizing forces—biomass surcharge loading^34^^,^^35^ and concentrated infiltration via stemflow.^36^^,^^37^ Using the Wu-Waldron root reinforcement model,^34^^,^^57^ published field measurements indicate root-enhanced cohesion (*C*_*r*_) values of 5–25 kPa,^15^^,^^17^^,^^58^ while surcharge loading from moderately dense canopies can impose 2–10 kPa on hillslopes.^35^^,^^59^ The CTZ thus corresponds to a transitional stage where these opposing forces are of comparable magnitude. We emphasize that this force-balance account remains a working hypothesis at the global scale: the published *C*_*r*_ and surcharge ranges overlap, and their *net* effect depends on biomass, rooting depth, and soil-moisture state, which satellite indices constrain only indirectly; a full mechanistic closure requires linking NDVI to site-level above-ground biomass and root cohesion through allometric and pull-out measurements. This perspective connects directly to physically based slope-stability theory: in the infinite-slope factor-of-safety framework, root reinforcement enters as an apparent-cohesion term, while canopy interception and stemflow modulate the rainfall-infiltration and pore-pressure dynamics that govern failure.^29^^,^^30^ The vegetation paradox can then be read as a regime in which the vegetation-dependent cohesion gain and the vegetation-dependent infiltration-and-loading penalty cross over within the CTZ density band.

### Predictive validation and the role of terrain

To address potential circularity in our predictive validation, we developed two complementary modeling frameworks (Tables S8–S12). First, a Random Forest model achieved spatial cross-validated *R*^2^ = 0.712 (root-mean-square error, RMSE = 0.171; Table S12). Its training *R*^2^ was higher (0.97), as is typical for Random Forests, but the substantial and positive cross-validated value—the appropriate generalization metric—confirms that the relationship generalizes beyond the training data rather than reflecting overfitting. Second, a fully independent grid-based validation (0.5°×0.5° cells, *n* = 364) predicting log landslide counts achieved *R*^2^ = 0.446 (RMSE = 1.088; Table S8), with mean EVI and mean NDVI among the leading predictors (Table S9). Its lower *R*^2^ follows from the small aggregated sample (364 cells) rather than from a weak vegetation signal; the diagnostic outcome is its partial-dependence function for mean NDVI, which rises to a maximum at 0.860 (Table S10), within the CTZ—independently locating this rising-limb maximum through a method that does not rely on histogram binning. The decline above the CTZ that completes the unimodal shape is evident in the raw landslide-frequency distribution itself (Figure 1B), which is computed without any background model and falls sharply above NDVI ≈0.89; the grid model does not resolve this declining limb, because aggregation compresses the upper vegetation tail: although cell-mean NDVI reaches a maximum of 0.921, only 18 of the 364 cells (5%) exceed 0.860, and the partial-dependence function is therefore evaluated over the central 5th–95th percentile range of the predictor (0.354–0.859; Table S10). Its maximum consequently coincides with the upper end of the evaluated range rather than with a turning point.

We directly tested whether topographic controls could explain the vegetation paradox by incorporating SRTM-derived terrain variables (slope, elevation, terrain ruggedness index [TRI], topographic wetness index [TWI], and topographic position index [TPI]) into the model. Including terrain produced only a marginal improvement (*R*^2^ = 0.717 vs. 0.712; Δ*R*^2^ = +0.005; Table S12), with slope ranked 10th of 14 predictors (Table S11). This small marginal contribution does not contradict the established primacy of slope in landslide susceptibility^60^: because our inventory consists exclusively of locations that have already failed, the range of slope among events is restricted (a case-only design), so slope retains little *residual* power to discriminate among vegetation-density classes even though it remains a first-order control on where landslides occur. The same case-only reasoning extends to the other terrain covariates (elevation, TRI, TWI, and TPI), whose among-event ranges are similarly restricted; the small terrain Δ*R*^2^ = +0.005 therefore reflects residual, within-inventory discriminative power rather than the absolute role of terrain in landslide occurrence. Consistent with this scale- and design-dependence, recent work integrating InSAR and machine learning has found soil type to outweigh slope and aspect for rainfall-triggered landslide susceptibility.^61^ A slope-stratified analysis further showed CTZ proportions of 16.7%, 35.5%, 44.5%, and 40.3% in the <15°, 15°–30°, 30°–45°, and >45° classes (Table S13)—demonstrating that the CTZ intensifies on steeper terrain rather than being a low-slope artifact.

## Implications for hazard mapping and land management

This framework informs landslide hazard management primarily through susceptibility mapping rather than temporal forecasting. At the global and regional scales, the CTZ thresholds allow systematic identification of high-priority monitoring areas; the global generality of vegetation-landslide associations and their continental heterogeneity are increasingly documented by large-sample machine-learning studies.^62^ We caution, however, against reading pre-failure NDVI dynamics as an operational early-warning signal: the apparent terminal decline does not survive correction for post-failure compositing contamination (Figure S10; Table S6), so satellite NDVI is better suited to delineating where susceptibility is elevated than to forecasting when a slope will fail—a task for which rainfall thresholds and InSAR-based deformation monitoring, with their 3–5 days rainfall-deformation lags,^63^ remain the appropriate tools. Our findings also carry policy implications: afforestation and ecological-restoration programs may, as a space-for-time hypothesis, create transient hazard windows if maturing stands pass through the CTZ density range^64^^,^^65^^,^^66^—a possibility that requires longitudinal monitoring of vegetation succession on restored slopes to confirm, rather than the cross-sectional inference available here.

Our analysis establishes that the relationship between vegetation and landslide susceptibility is fundamentally non-monotonic. The discovery of the “vegetation paradox” and its underlying mechanisms challenges a decades-old assumption in slope stability science. By providing objectively defined, globally calibrated vegetation-density thresholds, we open new pathways for satellite-based susceptibility mapping and a quantitative, threshold-based framework for global landslide hazard reduction.

### Limitations of the study

Several limitations qualify these findings. Our inventory is specific to rainfall-triggered landslides and may carry reporting biases toward populated areas.^67^ Nearly a third of events (30.9%) lacked reliable NDVI—a structural consequence of the trigger itself, since the monsoonal cloud that delivers the rainfall is opaque to all passive optical sensors, making the gap non-random and concentrated in the wettest monsoon terrain (95.5% of the 5,569 excluded events are flagged cloud-contaminated; STAR Methods); crucially, the breakpoints are essentially unchanged across data-quality (QA = 0), continental, and monsoon-Asia-excluded subsets (Figure S8; Table S3), the reported 23.0% CTZ share is therefore best read as a lower bound, and cloud-penetrating SAR (e.g., Sentinel-1) is the priority next step. We make no early-warning claim because the high-NDVI seasonal signal (Figure 3C) partly reflects rainfall seasonality and the terminal pre-failure NDVI window is contaminated by the post-failure scar (Figure S10; Table S6); the cross-sectional susceptibility result is nonetheless unaffected, as the breakpoints recover from every clean window. Our analysis characterizes the vegetation-density distribution of where rainfall-triggered failures occur rather than normalizing it by the land area available at each NDVI; the intermediate-density maximum is therefore best read as a robust property of the global landslide inventory—supported by its recovery within individual climate zones, land-cover classes, and slope bands (Figures 2 and S7; Table S13)—rather than a formally exposure-normalized susceptibility, and a background-normalized susceptibility analysis is an important direction for future work. Finally, terrain adds negligible predictive power (Δ*R*^2^ = +0.005; Table S12) while the CTZ intensifies on steeper slopes, and soil, lithology, and combined natural-anthropogenic drivers^68^^,^^69^^,^^70^ may further modulate the pattern; future work should integrate high-resolution soil data and test species-specific and longitudinal responses.^71^^,^^72^^,^^73^^,^^74^^,^^75^^,^^76^

## Resource availability

### Lead contact

Further information and requests for resources should be directed to and will be fulfilled by the lead contact, Shaoxiong Yuan (yuanshx@gdas.ac.cn).

### Materials availability

This study did not generate new unique materials.

### Data and code availability

• All landslide data have been archived at Zenodo with a permanent DOI (https://doi.org/10.5281/zenodo.21190727) and are publicly available as of the date of publication; the actively maintained development repository is at https://github.com/v4-hub/LanSVe. The DOI is listed in the key resources table.
• All original code has been archived at Zenodo with a permanent DOI (https://doi.org/10.5281/zenodo.21190727) and is publicly available as of the date of publication (development repository: https://github.com/v4-hub/LanSVe). The DOI is listed in the key resources table.
• Any additional information required to reanalyze the data reported in this paper is available from the lead contact upon request.

## Acknowledgments

This research was supported by the 10.13039/501100001809National Natural Science Foundation of China (grant no. 42271091), and the 10.13039/501100003453Guangdong Provincial Natural Science Foundation (grant nos. 2025A1515510020, 2024A1515510028 and 2024A1515030114). We acknowledge NASA for the Global Landslide Catalog, ESA for WorldCover and Copernicus products, USGS for the Hansen dataset, and the Google Earth Engine team. We are especially grateful to the anonymous reviewer, whose careful and constructive assessment—most notably the question on pre-failure trajectories that led us to identify and correct a compositing artefact—substantially strengthened the rigor and objectivity of this work.

## Author contributions

S.Y. conceived the study, performed the analysis, and wrote the original draft. Q.W. guided remote sensing methodology and revised the manuscript. Q.G. supervised the project and acquired funding. M.A.G.R. provided technical support for remote sensing and ML. G.S. advised on statistical methods. Y.Z. contributed to writing. Y.H., J.W., and Y.C. assisted with data compilation.

## Declaration of interests

The authors declare no competing interests.

## Declaration of generative AI and AI-assisted technologies in the writing process

During the preparation of this work, the author(s) used Claude (Anthropic) to assist with code debugging and optimization, and manuscript language polishing. The author(s) also used Perplexity AI to assist with preliminary literature searching. Because AI-assisted literature tools can generate inaccurate or fabricated citations, every reference in this manuscript was subsequently verified manually by the authors against the original published source and its registered DOI (via Crossref); no citation relies on AI-generated bibliographic information. After using these tools, the author(s) reviewed and edited all content and take(s) full responsibility for the content of the published article.

## STAR★Methods

### Key resources table

**Table:** 

REAGENT or RESOURCE	SOURCE	IDENTIFIER
**Deposited data**		

NASA Global Landslide Catalog (GLC/COOLR); *n* = 12,956, 2000–2024	Kirschbaum et al.^77^	https://gpm.nasa.gov/landslides/
e-ITALICA rainfall-induced landslide catalog; *n* = 4,599, 2000–2021	Brunetti et al.^78^	https://doi.org/10.1038/s41597-025-04551-6
GGIG Catalog (this paper); *n* = 473, 2017–2023	Guangzhou Institute of Geography, GDAS	Available from the lead contact
MODIS MOD13Q1 NDVI/EVI, 250 m, 16-day	NASA LP DAAC	https://lpdaac.usgs.gov/products/mod13q1v061/
MODIS MOD15A2H LAI/FPAR, 500 m, 8-day	NASA LP DAAC	https://lpdaac.usgs.gov/products/mod15a2hv061/
MODIS MCD12Q1 IGBP land cover	NASA LP DAAC	https://lpdaac.usgs.gov/products/mcd12q1v061/
Hansen Global Forest Change	Hansen et al.^79^	https://glad.earthengine.app/
Copernicus CGLS-LC100 land cover	Copernicus	https://land.copernicus.eu/
SRTM 30 m DEM	USGS	https://earthexplorer.usgs.gov/
MERIT Hydro (TWI)	Yamazaki et al.^80^	http://hydro.iis.u-tokyo.ac.jp/∼yamadai/MERIT_Hydro/
Köppen-Geiger climate zones	Beck et al.^81^	http://www.gloh2o.org/koppen/
Curated landslide inventory and analysis outputs (this paper)	This paper	Zenodo: https://doi.org/10.5281/zenodo.21190727

**Software and algorithms**		

R v4.3.0	R Core Team	RRID:SCR_001905
segmented (R package)	Muggeo	https://CRAN.R-project.org/package=segmented
randomForest (R package)	Liaw and Wiener^82^	https://CRAN.R-project.org/package=randomForest
scikit-learn (Python)	Pedregosa et al.^83^	RRID:SCR_002577
mgcv (R package)	Wood	https://CRAN.R-project.org/package=mgcv
Google Earth Engine	Google	RRID:SCR_019964
Python 3.x	Python Software Foundation	RRID:SCR_008394
Analysis code (this paper)	This paper	Zenodo: https://doi.org/10.5281/zenodo.21190727; GitHub: https://github.com/v4-hub/LanSVe

### Method details

#### Landslide inventory and data preprocessing

Our study is based on a global database of rainfall-triggered landslide events compiled from three authoritative sources: the NASA Global Landslide Catalog/Cooperative Open Online Landslide Repository (GLC/COOLR, *n* = 12,956; 2000–2024),^77^ the enhanced ITAlian rainfall-induced LandslIdes Catalog (e-ITALICA, *n* = 4,599; 2000–2021),^78^ and a regional catalog compiled by the Guangzhou Institute of Geography, Guangdong Academy of Sciences (GGIG Catalog, *n* = 473; 2017–2023). The NASA catalog contains events of multiple trigger types; we retained only rainfall-triggered events—those whose trigger attribute denoted rain, heavy rain, downpour, monsoon, or continuous rainfall—and excluded seismic, snowmelt, anthropogenic, and unknown-trigger events. After this trigger filtering and a rigorous quality-control protocol, the curated dataset comprised 18,028 events spanning 2000–2024, all of which satisfy the rainfall-trigger criterion; this curated inventory, together with its extracted vegetation-index, land-cover, and terrain attributes, is provided as Data S1. Our primary analysis focused on the subset of 12,459 events (69.1%) with reliable pre-failure MODIS NDVI data. Of the remaining 5,569 events without reliable NDVI, 5,317 (95.5%) carried the MODIS MOD13Q1 VI-quality cloud state (QA = 3), quantifying the cloud origin of the gap. The GGIG Catalog comprises rainfall-triggered landslides in southern China (Guangdong) that we compiled and curated from official geological-hazard monitoring records obtained through a research collaboration (key resources table). In the regional disaster-reporting workflow, each landslide—a damaging event that receives careful attention at every level—is reported upward through successive local administrative levels (village, township, and county) with on-site photographic documentation, and professional geological teams additionally conduct field verification on a sampled (proportional) basis, exhaustive point-by-point field checking being cost-prohibitive; from these records we retained only events with usable coordinates and dates and an unambiguous rainfall trigger. The GGIG Catalog represents only 2.6% of the total inventory (catalog metadata in Table S14).

Quality control involved: (1) removing events lacking precise coordinates or dates; (2) excluding duplicates identified by spatial (<100 m) and temporal (<7 days) proximity; (3) eliminating events with low source reliability; and (4) filtering events in implausible locations.

#### Geospatial data extraction

All geospatial data were extracted using Google Earth Engine (GEE). For each event, we extracted vegetation indices for five 16-day windows preceding the event (0–16, 16–32, 32–48, 48–64, and 64–80 days). Because MOD13Q1 is itself a 16-day composite, the nearest (0–16 days) window can incorporate observations acquired after the failure date: the composite assigned to this terminal window begins a median of 7 days before failure and spans a median of 9 days *after* it, so for 96% of events it includes the post-failure surface, including the bare-soil landslide scar. We therefore quantify this contamination and exclude the affected window when testing for pre-failure trajectories (Results; Figure S10; Table S6). Primary indices included NDVI and EVI from MODIS (MOD13Q1 V6.1, 250 m), and LAI/FPAR from MODIS (MOD15A2H V6.1, 500 m). Land cover was derived from MODIS (MCD12Q1), Copernicus (CGLS-LC100), and Hansen datasets. Climate zones were from the Köppen-Geiger classification.^81^ Terrain variables (elevation, slope, aspect, TPI, TRI, curvature, TWI) were extracted from SRTM 30 m DEM and MERIT Hydro.

#### Statistical analysis

All analyses were performed in R (v4.3.0).^84^ Temporal trends used the Mann-Kendall test with Sen’s slope estimation (zone-level results in Table S15). Group comparisons used the Kruskal-Wallis test with Dunn’s post-hoc pairwise comparisons (Bonferroni-corrected). Seasonal distributions were compared using Chi-squared tests.

#### Data-driven threshold detection

Breakpoints were identified using the segmented R package.^85^ The landslide-frequency distribution along the NDVI axis was constructed by binning NDVI into 150 equal-width bins and applying Tukey running-median smoothing (3R–S–3R); a two-breakpoint segmented model was then fitted:

$$y=\beta_{0}+\beta_{1}x+\beta_{2}{\left(x-\psi_{1}\right)}^{+}+\beta_{3}{\left(x-\psi_{2}\right)}^{+}+\varepsilon$$

where *y* is smoothed landslide frequency, *x* is NDVI, and (·)^+^ denotes the positive part. Starting breakpoint values *ψ* were initialized adaptively from the distribution shape and constrained to the interior 90% of the observed NDVI range; for the primary NDVI result the smoothed distribution was classified as neither strictly single-peaked nor single-valleyed by this heuristic, so *ψ* was initialized at the 0.25 and 0.75 quantiles of the binned NDVI distribution (quantile-based). Because the segmented algorithm refines the breakpoints iteratively, the estimates (BP1 = 0.769, BP2 = 0.868) are insensitive to these starting values, consistent with the binning- and bandwidth-robustness in Table S4. Bootstrap confidence intervals (10,000 iterations, BCa) were computed by resampling the smoothed distribution. Breakpoint robustness was assessed against the number of bins (75–200) and Gaussian kernel-density bandwidths (Silverman and Scott rules, scaled 0.5×–2×; Table S4), against the MODIS cloud-QA threshold (Table S3), and against simulated sensor noise (*σ* up to 0.05; Table S5). Independent validation used GAMs with thin-plate splines.^86^

#### Predictive modeling

The pixel-level Random Forest (R randomForest package^82^; ntree = 200 for cross-validation and 500 for the final model; default mtry =⌊*p*/3⌋ for regression) used 5-fold spatially stratified (latitudinal-band) cross-validation with 9 predictor variables (excluding primary NDVI). We report both training and cross-validated *R*^2^ to assess overfitting (Table S12). A second, fully independent grid-based validation (Python scikit-learn RandomForestRegressor^83^; n_estimators = 200, max_depth = 10, min_samples_leaf = 5) aggregated events into 0.5°×0.5° cells (*n* = 364), predicting log-transformed landslide counts. Terrain robustness was tested by adding 5 SRTM-derived variables and comparing feature importance and Δ*R*^2^.

The grid-based Random Forest (above) predicts landslide counts per fixed-area 0.5° cell and provides a binning-free check on the location of the frequency maximum. Because the grid is constructed from landslide locations only, it does not normalize for the land area available at each NDVI; we treat this availability normalization as a limitation (see limitations of the study). Spatial autocorrelation was assessed using Moran’s *I*^87^ on a random subsample of 5,000 events (Table S16).

### Quantification and statistical analysis

All statistical analyses were performed in R v4.3.0,^84^ except the grid-based Random Forest, which used Python scikit-learn.^83^ Statistical parameters, including the sample size *n*, the specific test used, and the significance threshold, are reported in the Results, the figure legends, and the supplemental tables. Throughout, *n* refers to the number of individual landslide events, except in the grid-based validation, where *n* is the number of 0.5° grid cells. Two-sided tests were used and *p* < 0.05 was considered statistically significant; exact *p* values are reported except where *p* < 0.001.

Breakpoints in the smoothed landslide-frequency distribution were estimated by two-breakpoint segmented regression,^85^ with fit quality summarized by the coefficient of determination, the adjusted *R*^2^, and the residual standard error on the stated degrees of freedom. Uncertainty in the breakpoints is reported as 95% bias-corrected and accelerated (BCa) bootstrap confidence intervals from 10,000 resamples (Figure S1; Table S1); these intervals are the red bands in Figure 1B. Differences in pre-failure NDVI among Köppen climate zones were tested with the Kruskal-Wallis rank-sum test, followed by Dunn’s post-hoc pairwise comparisons with Bonferroni correction (Figure 3B). Seasonal distributions of failure timing were compared with Chi-squared tests (Figure 3C). Within-event pre-failure NDVI changes were tested with paired tests by zone (Tables S6 and S7). Temporal trends used the Mann-Kendall test with Sen’s slope estimator (Table S15). Random Forest performance is reported as the cross-validated *R*^2^ and RMSE under 5-fold spatially stratified cross-validation, with training values given alongside for the overfitting assessment (Table S12). Boxplots display the median (center line), the interquartile range (box), whiskers extending to at most 1.5× the interquartile range, and individual points beyond that range; no data were excluded as outliers. No statistical comparisons are annotated with asterisks in any figure.

## References

[1] Froude M.J., Petley D.N. (2018). Global fatal landslide occurrence from 2004 to 2016. Nat. Hazards Earth Syst. Sci..

[2] Petley D. (2012). Global patterns of loss of life from landslides. Geology.

[3] Oxera (2024). The Economic Cost of Extreme Weather Events — Prepared for the International Chamber of Commerce.

[4] Petrucci O. (2022). Landslide fatality occurrence: A systematic review of research published between January 2010 and March 2022. Sustainability.

[5] Vries M. de, Dunant A., Johnson A.L., Harvey E.L., Li S., Arrell K., Baniya J., Basnet D., Basyal G.K., Rosser N.J. (2025). Brief communication: Weak correlation between building damage and loss of life from landslides. Nat. Hazards Earth Syst. Sci..

[6] Silva R.C.d., Mendes R.M., Fisch G. (2020). Future scenarios (2021-2050) of extreme precipitation events that trigger landslides – a case study of the paraitinga river watershed, SP, Brazil. Rev. Ambient. e Agua.

[7] Cao Z., Hu S., Tao Z. (2023). Analyze comments on Twitter about extreme weather based on the Latent Dirichlet Allocation (LDA) approach. Proc. SPIE.

[8] Lee H., Romero J., Core Writing Team (2023). Climate change 2023: Synthesis report. Contribution of working groups I, II and III to the sixth assessment report of the intergovernmental panel on climate change.

[9] Gariano S.L., Guzzetti F. (2016). Landslides in a changing climate. Earth Sci. Rev..

[10] Guzzetti F., Carrara A., Cardinali M., Reichenbach P. (1999). Landslide hazard evaluation: A review of current techniques and their application in a multi-scale study, Central Italy. Geomorphology.

[11] Norris J.E., Stokes A., Mickovski S.B., Cammeraat E., Beek R., Nicoll B.C., Achim A. (2008). Slope stability and erosion control: Ecotechnological solutions. Proc. Inst. Civ. Eng. - Eng. Sustain..

[12] Cohen-Shacham E., Walters G., Janzen C., Maginnis S. (2016).

[13] Stokes A., Atger C., Bengough A.G., Fourcaud T., Sidle R.C. (2009). Desirable plant root traits for protecting natural and engineered slopes against landslides. Plant Soil.

[14] Gonzalez-Ollauri A., Mickovski S.B. (2017). Plant-soil reinforcement response under different soil hydrological conditions. Geoderma.

[15] Schwarz M., Preti F., Giadrossich F., Lehmann P., Or D. (2010). Quantifying the role of vegetation in slope stability: A case study in Tuscany (Italy). Ecol. Eng..

[16] Giadrossich F., Schwarz M., Cohen D., Preti F., Or D. (2013). Mechanical interactions between neighbouring roots during pullout tests. Plant Soil.

[17] Docker B.B., Hubble T.C.T. (2008). Quantifying root-reinforcement of river bank soils by four Australian tree species. Geomorphology.

[18] Van Dijk A.I.J.M., Keenan R.J. (2007). Planted forests and water in perspective. For. Ecol. Manage..

[19] Keim R.F., Skaugset A.E. (2003). Modelling effects of forest canopies on slope stability. Hydrol. Process..

[20] Sidle R.C., Bogaard T.A. (2016). Dynamic earth system and ecological controls of rainfall-initiated landslides. Earth Sci. Rev..

[21] Bogaard T., Greco R. (2018). Invited perspectives: Hydrological perspectives on precipitation intensity-duration thresholds for landslide initiation: Proposing hydro-meteorological thresholds. Nat. Hazards Earth Syst. Sci..

[22] Stanley T., Kirschbaum D.B. (2017). A heuristic approach to global landslide susceptibility mapping. Nat. Hazards.

[23] Pourghasemi H.R., Yansari Z.T., Panagos P., Pradhan B. (2018). Analysis and evaluation of landslide susceptibility: A review on articles published during 2005–2016. Arab. J. Geosci..

[24] Merghadi A., Yunus A.P., Dou J., Whiteley J., ThaiPham B., Bui D.T., Avtar R., Abderrahmane B. (2020). Machine learning methods for landslide susceptibility studies: A comparative overview of algorithm performance. Earth Sci. Rev..

[25] Samia J., Temme A., Bregt A., Wallinga J., Guzzetti F., Ardizzone F., Rossi M. (2017). Characterization and quantification of path dependency in landslide susceptibility. Geomorphology.

[26] Tanyaş H., van Westen C.J., Allstadt K.E., Anna Nowicki Jessee M., Görüm T., Jibson R.W., Godt J.W., Sato H.P., Schmitt R.G., Marc O., Hovius N. (2017). Presentation and analysis of a worldwide database of earthquake-induced landslide inventories. JGR. Earth Surface.

[27] Chen W., Pourghasemi H.R., Zhao Z. (2017). A GIS-based comparative study of Dempster-Shafer, logistic regression and artificial neural network models for landslide susceptibility mapping. Geocarto Int..

[28] Goetz J.N., Brenning A., Petschko H., Leopold P. (2015). Evaluating machine learning and statistical prediction techniques for landslide susceptibility modeling. Comput. Geosci..

[29] Iverson R.M. (2000). Landslide triggering by rain infiltration. Water Resour. Res..

[30] Lu N., Godt J. (2008). Infinite slope stability under steady unsaturated seepage conditions. Water Resour. Res..

[31] Sidle R.C., Ochiai H. (2006).

[32] Glade T. (2005). Linking debris-flow hazard assessments with geomorphology. Geomorphology.

[33] Keefer D.K. (2002). Investigating landslides caused by earthquakes – a historical review. Surv. Geophys..

[34] Wu T.H., McKinnell W.P., Swanston D.N. (1979). Strength of tree roots and landslides on Prince of Wales Island, Alaska. Can. Geotech. J..

[35] Schmidt K.M., Roering J.J., Stock J.D., Dietrich W.E., Montgomery D.R., Schaub T. (2001). The variability of root cohesion as an influence on shallow landslide susceptibility in the Oregon Coast Range. Can. Geotech. J..

[36] Sidle R.C., Ziegler A.D. (2017). The canopy interception-landslide initiation conundrum: Insight from a tropical secondary forest in northern Thailand. Hydrol. Earth Syst. Sci..

[37] Johnson A.C., Wilcock P. (2002). Association between cedar decline and hillslope stability in mountainous regions of southeast Alaska. Geomorphology.

[38] Meunier P., Hovius N., Haines J.A. (2008). Topographic site effects and the location of earthquake induced landslides. Earth Planet Sci. Lett..

[39] Corominas J., Moya J. (2008). A review of assessing landslide frequency for hazard zoning purposes. Eng. Geol..

[40] Handwerger A.L., Roering J.J., Schmidt D.A. (2013). Controls on the seasonal deformation of slow-moving landslides. Earth Planet Sci. Lett..

[41] Intrieri E., Carlà T., Fumagalli A., Lu P., Del Conte S., Farina P., Allievi J., Ferretti A., Casagli N. (2018). The Maoxian landslide as seen from space: Detecting precursors of failure with Sentinel-1 data. Landslides.

[42] Zhao C., Lu Z. (2018). Remote sensing of landslides—a review. Remote Sens..

[43] Uhlemann S., Smith A., Chambers J., Dixon N., Dijkstra T., Haslam E., Meldrum P., Merritt A., Gunn D., Mackay J. (2016). Assessment of ground-based monitoring techniques applied to landslide investigations. Geomorphology.

[44] Calvello M., Papa M.N., Pratschke J., Nacchia Crescenzo M. (2016). Landslide risk perception: A case study in Southern Italy. Landslides.

[45] Haque U., Blum P., da Silva P.F., Andersen P., Pilz J., Chalov S.R., Malet J.P., Auflič M.J., Andres N., Poyiadji E. (2016). Fatal landslides in Europe. Landslides.

[46] Guns M., Vanacker V. (2014). Shifts in landslide frequency–magnitude distribution after forest conversion in the tropical Andes. Anthropocene.

[47] Brenning A. (2005). Spatial prediction models for landslide hazards: Review, comparison and evaluation. Nat. Hazards Earth Syst. Sci..

[48] Carrara A., Cardinali M., Guzzetti F., Reichenbach P. (1995). GIS technology in mapping landslide hazard. Geogr. Inf. Syst. Assess. Nat. Hazards.

[49] Varnes D.J. (1978). Slope movement types and processes. Spec. Rep. 176 Landslides Anal. Control.

[50] Cruden D.M., Varnes D.J. (1996). Landslides: Investigation and mitigation. Chapter 3—Landslide types and processes. Transp. Res. Board Spec. Rep..

[51] Highland L.M., Bobrowsky P. (2008).

[52] Aleotti P., Chowdhury R. (1999). Landslide hazard assessment: Summary review and new perspectives. Bull. Eng. Geol. Environ..

[53] Guzzetti F., Reichenbach P., Cardinali M., Galli M., Ardizzone F. (2005). Probabilistic landslide hazard assessment at the basin scale. Geomorphology.

[54] Tian H., Kou P., Xu Q., Tao Y., Jin Z., Xia Y., Feng J., Liu R., Gou Y. (2024). Analysis of landslide deformation in eastern qinghai province, northwest china, using SBAS-InSAR. Nat. Hazards.

[55] Genet M., Stokes A., Salin F., Mickovski S.B., Fourcaud T., Dumail J.F., van Beek R. (2005). The influence of cellulose content on tensile strength in tree roots. Plant Soil.

[56] Fan C.-C., Su C.-F. (2008). Role of roots in the shear strength of root-reinforced soils with high moisture content. Ecol. Eng..

[57] Waldron L.J. (1977). The shear resistance of root-permeated homogeneous and stratified soil. Soil Sci. Soc. Am. J..

[58] Mao Z., Saint-André L., Genet M., Mine F.X., Jourdan C., Rey H., Courbaud B., Stokes A. (2012). Engineering ecological protection against landslides in diverse mountain forests: Choosing cohesion models. Ecol. Eng..

[59] Greenway D.R. (1987). Vegetation and slope stability. Slope Stab.

[60] Reichenbach P., Rossi M., Malamud B.D., Mihir M., Guzzetti F. (2018). A review of statistically-based landslide susceptibility models. Earth Sci. Rev..

[61] Yu H., Kou P., Xu Q., Yuan Z., Dong X., Liang W., Peng D., Tang M., Chen L., Pu C., Jin Z. (2026). Integrated InSAR and machine learning reveal soil type as primary control on rainfall-triggered landslide susceptibility in Meizhou, China. Land Degrad. Dev..

[62] Kou P., Yu H., Xu Q., Tang M., Yuan Z., Dong X., Li H., Pu C., Jin Z. (2026). Machine learning-based global landslide susceptibility analysis: Spatiotemporal variability and dominant environmental associations. Bull. Eng. Geol. Environ..

[63] Kou P., Tao Y., Cui J., Jin Z., Huang Y., Liang W., Alonso-Rodriguez A., Xu Q. (2026). Hydro-gravitational dominance and differential landslide deformation unveiled by SBAS-InSAR-enhanced susceptibility mapping in wushan, three gorges. Nat. Hazards.

[64] Capobianco V., Fraccica A., Anselmucci F., Tagarelli V. (2025). Nature- and bio-based solutions for ecosystem restoration, landslide hazard mitigation, and ground improvement: Research and application novelties. Ecol. Eng..

[65] Sudmeier-Rieux K., Masundire H., Rizvi A., Rietbergen S. (2006).

[66] Renaud F.G., Sudmeier-Rieux K., Estrella M., Nehren U. (2016).

[67] Guzzetti F., Mondini A.C., Cardinali M., Fiorucci F., Santangelo M., Chang K.T. (2012). Landslide inventory maps: New tools for an old problem. Earth Sci. Rev..

[68] Lombardo L., Mai P.M. (2018). Presenting logistic regression-based landslide susceptibility results. Eng. Geol..

[69] Steger S., Brenning A., Bell R., Petschko H., Glade T. (2016). Exploring discrepancies between quantitative validation results and the geomorphic plausibility of statistical landslide susceptibility maps. Geomorphology.

[70] Yang R., Liang W., Yu C., Kou P. (2024). Elucidating the complex interplay between natural and anthropogenic factors in the deformation of the muyubao landslide through time-series InSAR analysis. Front. Earth Sci..

[71] Tarolli P., Borga M., Chang K.-T., Chiang S.-H. (2011). Modeling shallow landsliding susceptibility by incorporating heavy rainfall statistical properties. Geomorphology.

[72] Catani F., Lagomarsino D., Segoni S., Tofani V. (2013). Landslide susceptibility estimation by random forests technique: Sensitivity and scaling issues. Nat. Hazards Earth Syst. Sci..

[73] Titti G., Borgatti L., Zou Q., Cui P., Pasuto A. (2021). Landslide susceptibility in the Belt and Road Countries: Continental step of a multi-scale approach. Environ. Earth Sci..

[74] Schlögel R., Marchesini I., Alvioli M., Reichenbach P., Rossi M., Malet J.P. (2018). Optimizing landslide susceptibility zonation: Effects of DEM spatial resolution and slope unit delineation on logistic regression models. Geomorphology.

[75] Aditian A., Kubota T., Shinohara Y. (2018). Comparison of GIS-based landslide susceptibility models using frequency ratio, logistic regression, and artificial neural network in a tertiary region of Ambon, Indonesia. Geomorphology.

[76] Tehrani F.S., Calvello M., Liu Z., Zhang L., Lacasse S. (2022). Machine learning and landslide studies: Recent advances and applications. Nat. Hazards.

[77] Kirschbaum D., Stanley T., Zhou Y. (2015). Spatial and temporal analysis of a global landslide catalog. Geomorphology.

[78] Brunetti M.T., Gariano S.L., Melillo M., Rossi M., Peruccacci S. (2025). An enhanced rainfall-induced landslide catalogue in Italy. Sci. Data.

[79] Hansen M.C., Potapov P.V., Moore R., Hancher M., Turubanova S.A., Tyukavina A., Thau D., Stehman S.V., Goetz S.J., Loveland T.R. (2013). High-resolution global maps of 21st-century forest cover change. Science.

[80] Yamazaki D., Ikeshima D., Sosa J., Bates P.D., Allen G.H., Pavelsky T.M. (2019). MERIT Hydro: a high-resolution global hydrography map based on latest topography dataset. Water Resour. Res..

[81] Beck H.E., Zimmermann N.E., McVicar T.R., Vergopolan N., Berg A., Wood E.F. (2018). Present and future Köppen-Geiger climate classification maps at 1-km resolution. Sci. Data.

[82] Liaw A., Wiener M. (2002). Classification and regression by randomForest. R. News.

[83] Pedregosa F., Varoquaux G., Gramfort A., Michel V., Thirion B., Grisel O., Blondel M., Prettenhofer P., Weiss R., Dubourg V. (2011). Scikit-learn: Machine learning in Python. J. Mach. Learn. Res..

[84] R Core Team (2023).

[85] Fasola S., Muggeo V.M.R., Küchenhoff H. (2018). A heuristic, iterative algorithm for change-point detection in abrupt change models. Comput. Stat..

[86] Wood S.N. (2017).

[87] Bivand R.S., Pebesma E., Gómez-Rubio V. (2013).

