# Supplementary material for: Global satellite analysis reveals landslide susceptibility peaks at intermediate vegetation density
Source: iScience. 2026 Aug 20;29(9):117271. doi: 10.1016/j.isci.2026.117271 (PMC13521072; doi:10.1016/j.isci.2026.117271)
Supplement: Supplementary file 1 [file mmc1.pdf]

iScience, Volume *29*

Supplemental information

Global satellite analysis reveals landslide susceptibility peaks at intermediate vegetation density

Shaoxiong Yuan, Quan Wang, Qinghua Gong, Miguel A. Gonzalez-Rodriguez, Guangman Song, Yongqiang Zong, Yinlei Hao, Jun Wang, and Yu Chen

Supplemental Figures

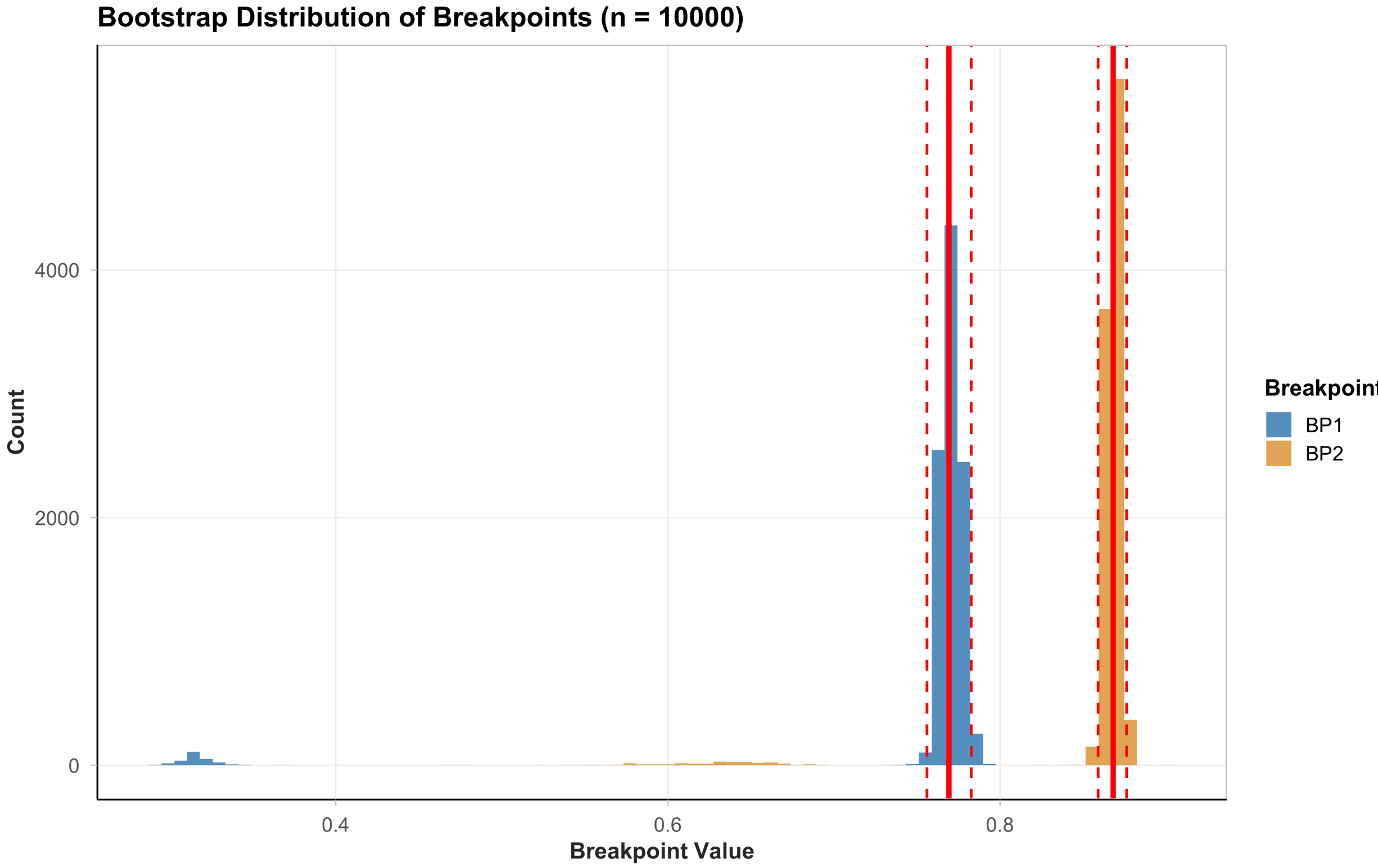

Figure S1. Bootstrap distribution and 95% BCa confidence intervals for the NDVI breakpoint estimates (10,000 resamples).

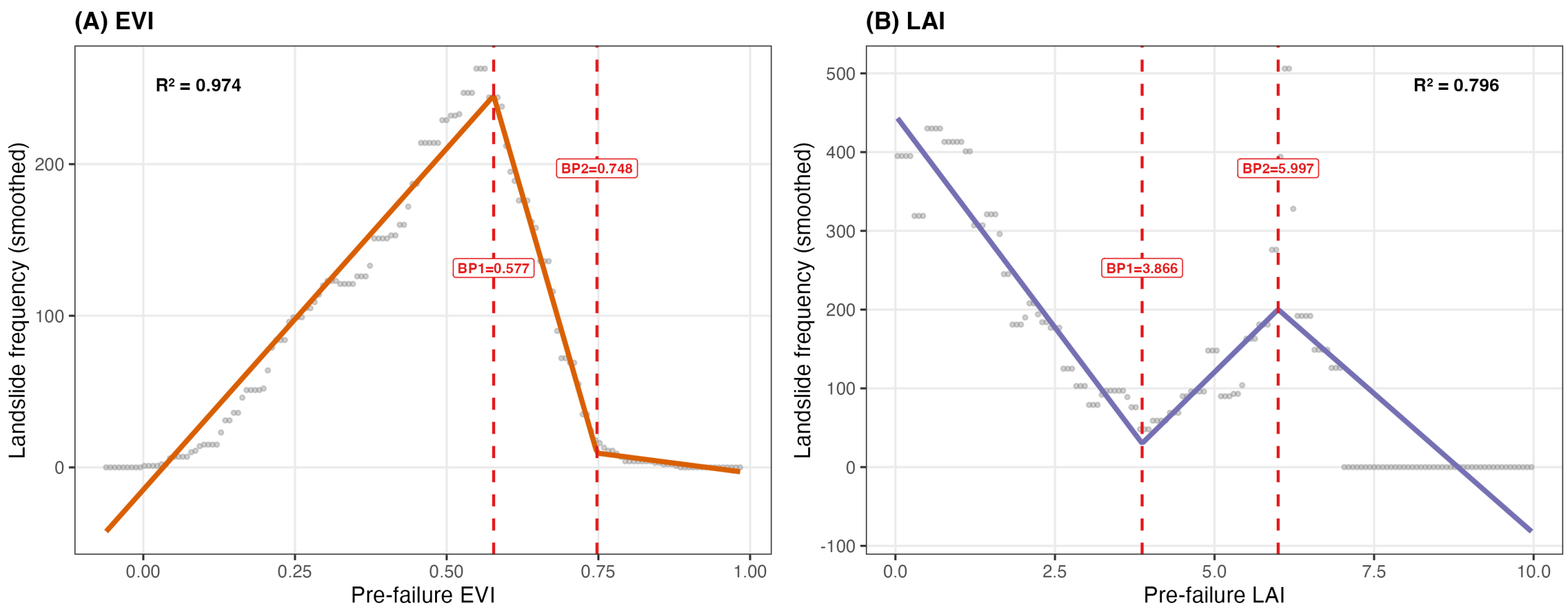

Figure S2. Segmented breakpoint analysis for EVI (BP1 = 0.577, BP2 = 0.748; R^2 = 0.974) and LAI (BP1 = 3.866, BP2 = 5.997; R^2 = 0.796), showing the intermediate-density susceptibility peak is reproduced by independent vegetation indices. The three indices differ in curve shape because NDVI (and, less severely, EVI) saturate once LAI $\gtrsim$ 3, compressing moderate-to-dense canopies into a narrow high-index band, whereas LAI resolves canopy structure across its full range. The breakpoints nonetheless coincide: events in the NDVI-defined IDZ ($<$0.769) have a median LAI of 1.1, CTZ events (0.769–0.868) a median of 4.7, and SDZ events ($>$0.868) a median of 6.0, so the NDVI thresholds correspond closely to the LAI breakpoints (3.866, 5.997), which bracket the same Critical Transition Zone in leaf-area units. The apparently bimodal LAI frequency curve reflects this saturation mapping: the low-LAI mode is the large, sparsely-vegetated instability-dominated population ($\sim$53% of events have LAI $<$ 3.9—spread thinly along the lower NDVI axis but concentrated at low LAI, where canopy and roots are too sparse to reinforce the soil), the secondary peak near LAI 6 is the CTZ (where root reinforcement is overtaken by biomass surcharge and stemflow-concentrated infiltration), and the decline beyond is the stability-dominated closed-canopy regime. LAI's lower R^2 is consistent with its coarser 500 m retrieval (MOD15A2H) and larger uncertainty over sparse canopies; NDVI is therefore used as the primary index, with EVI and LAI as independent cross-checks.

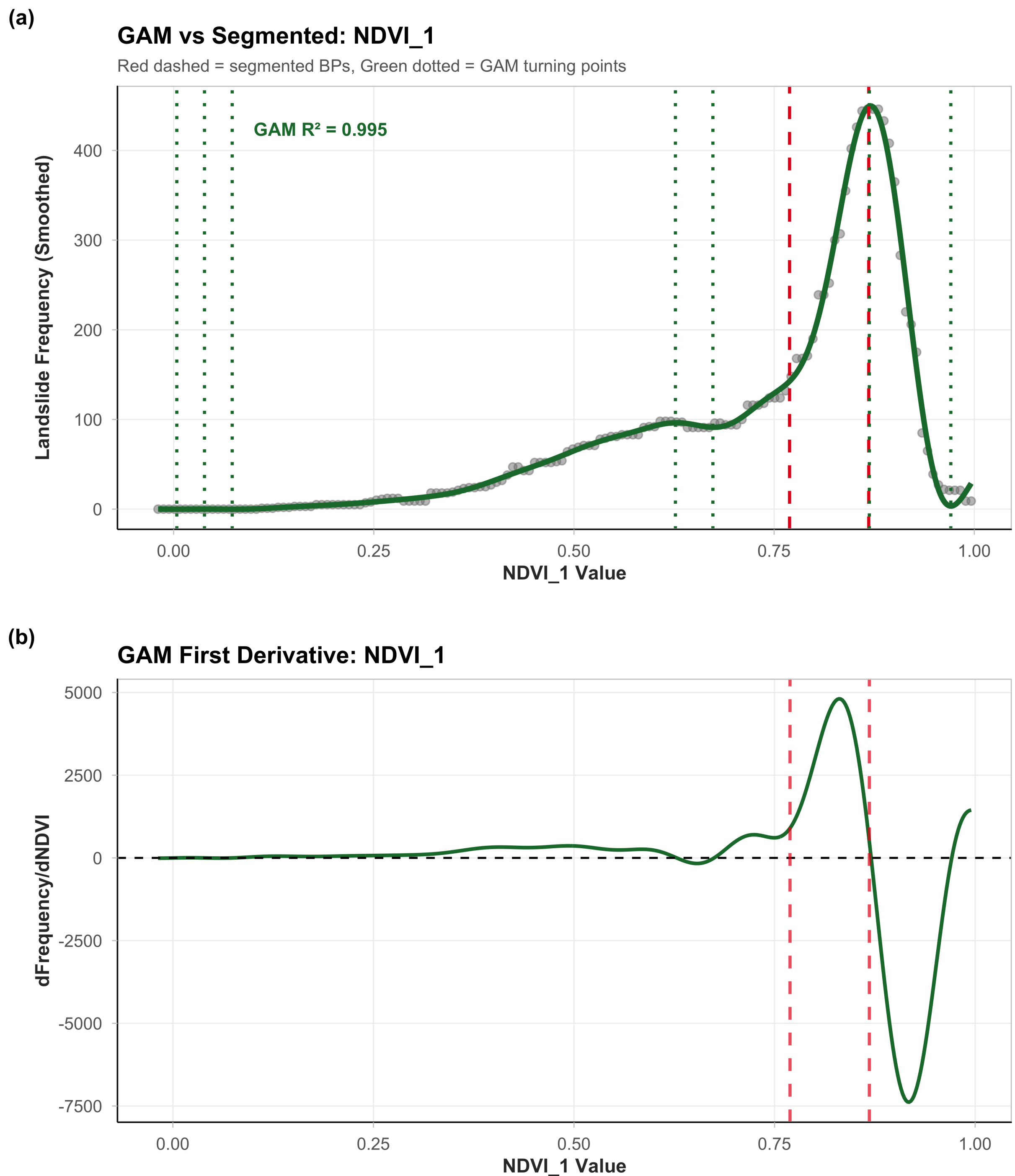

Figure S3. Generalized additive model (GAM) validation of the unimodal pattern using NDVI.

(a)

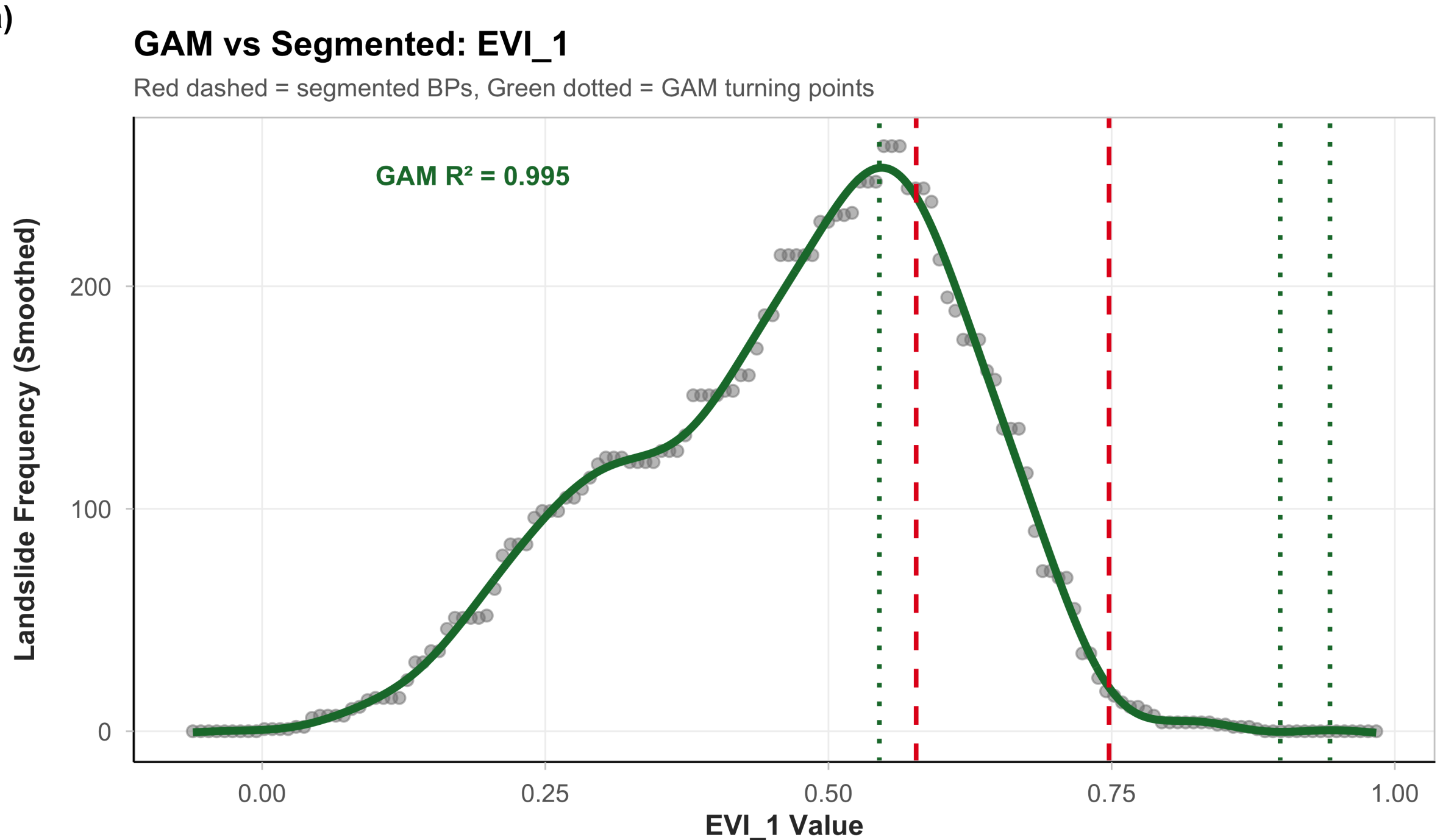

(b)

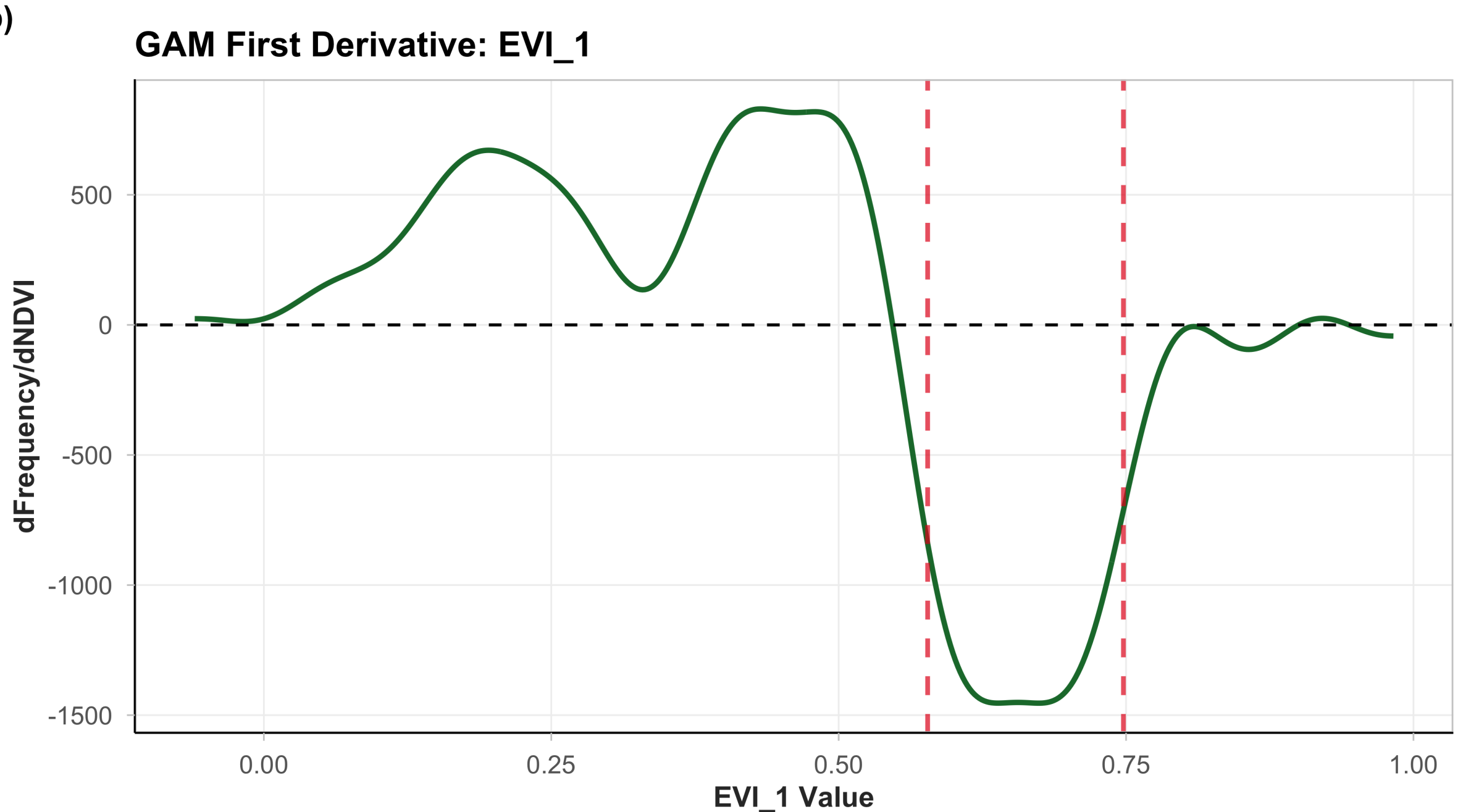

Figure S4. GAM validation using EVI.

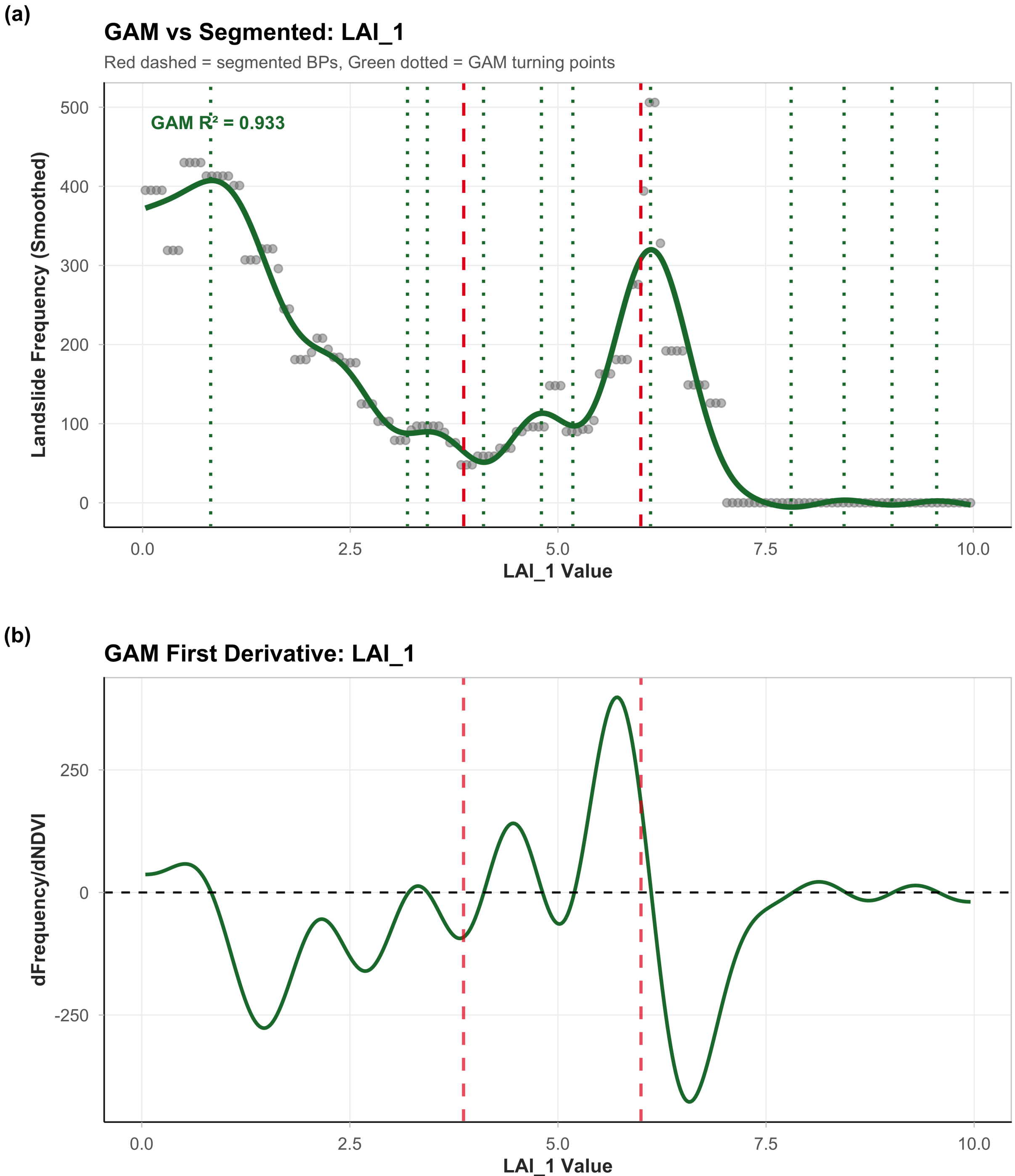

Figure S5. GAM validation using LAI.

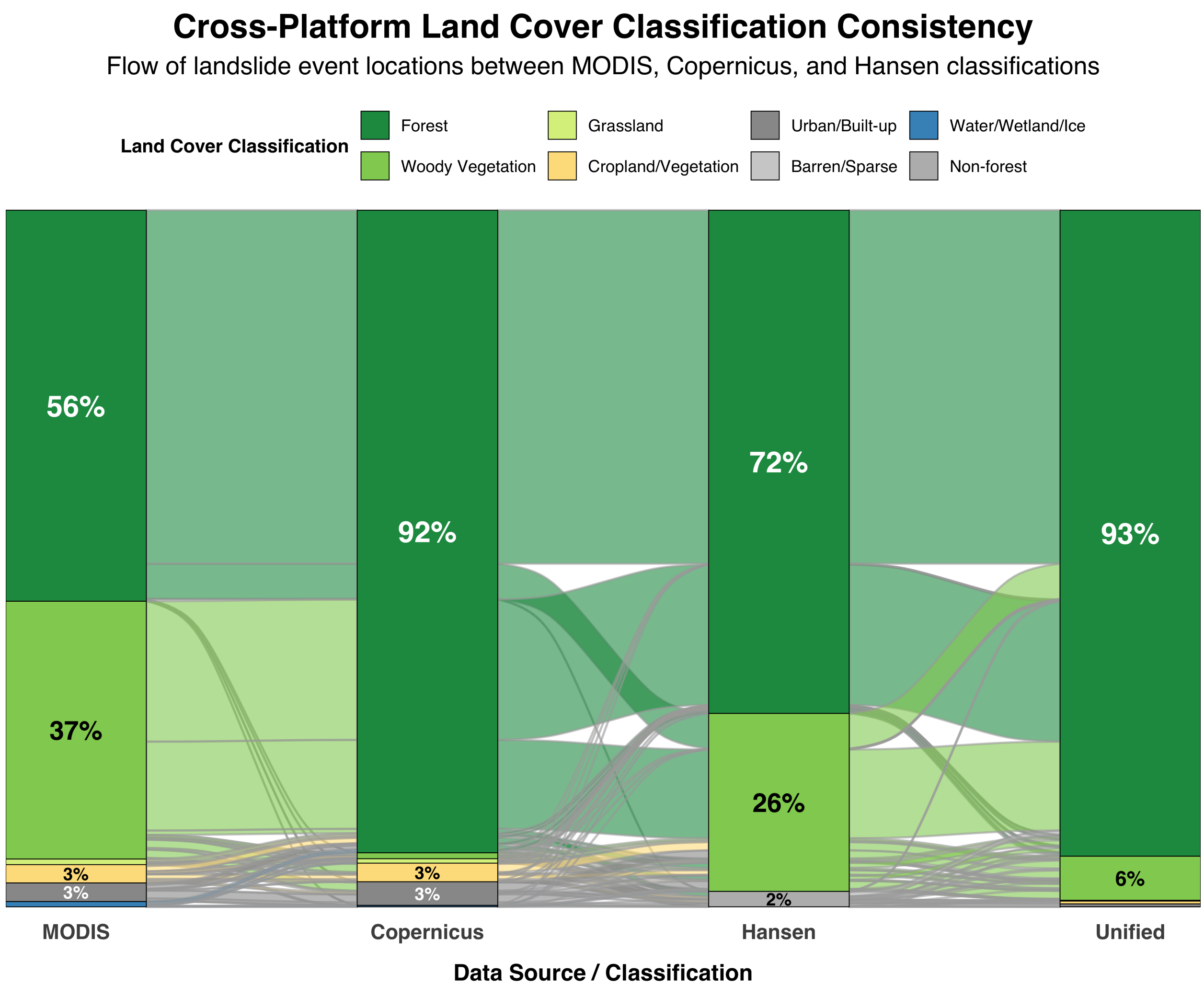

Figure S6. Sankey diagram of cross-platform land cover consistency (MODIS, Copernicus, Hansen).

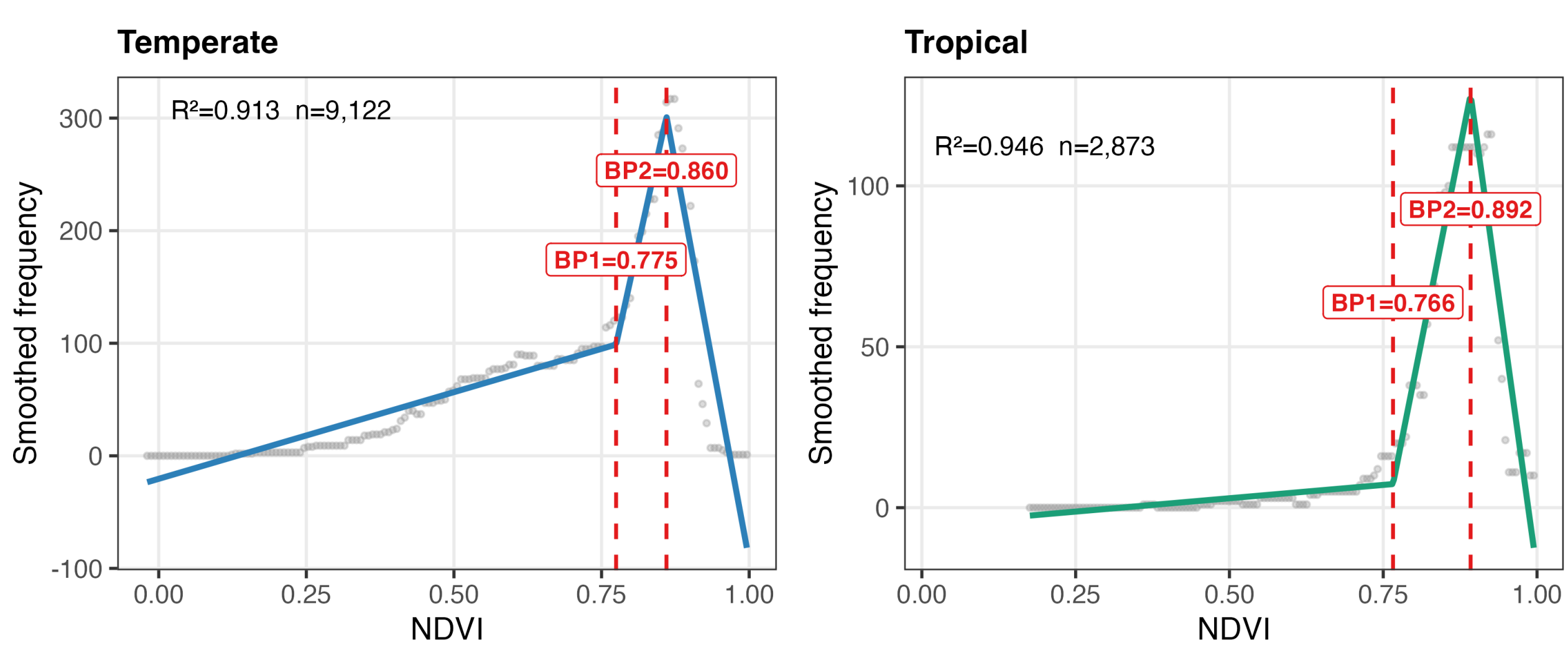

Figure S7. Faceted breakpoint analysis re-derived within individual climate zones.

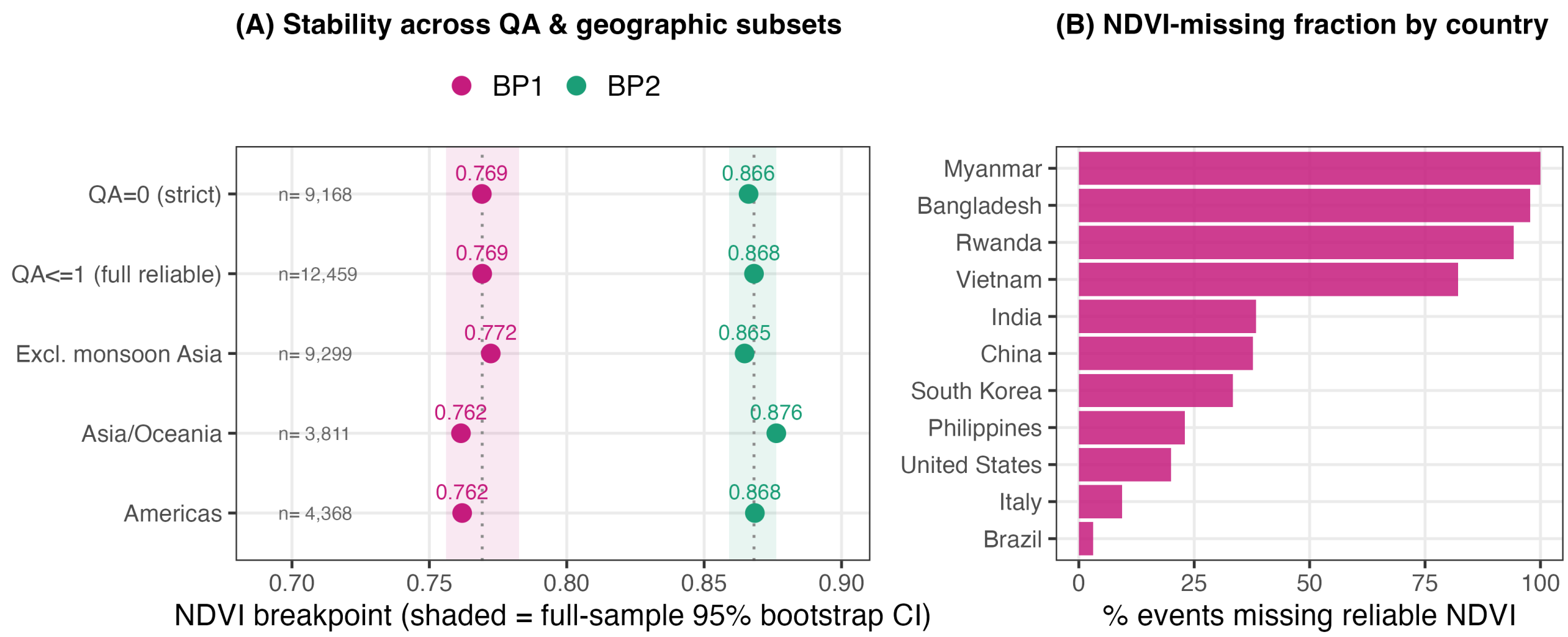

Figure S8. Missing-data robustness: NDVI breakpoints across data-quality (QA) and geographic subsets (by continent; monsoon Asia excluded) all fall within the full-sample bootstrap CI; and the cloud-coupled distribution of NDVI missingness by country.

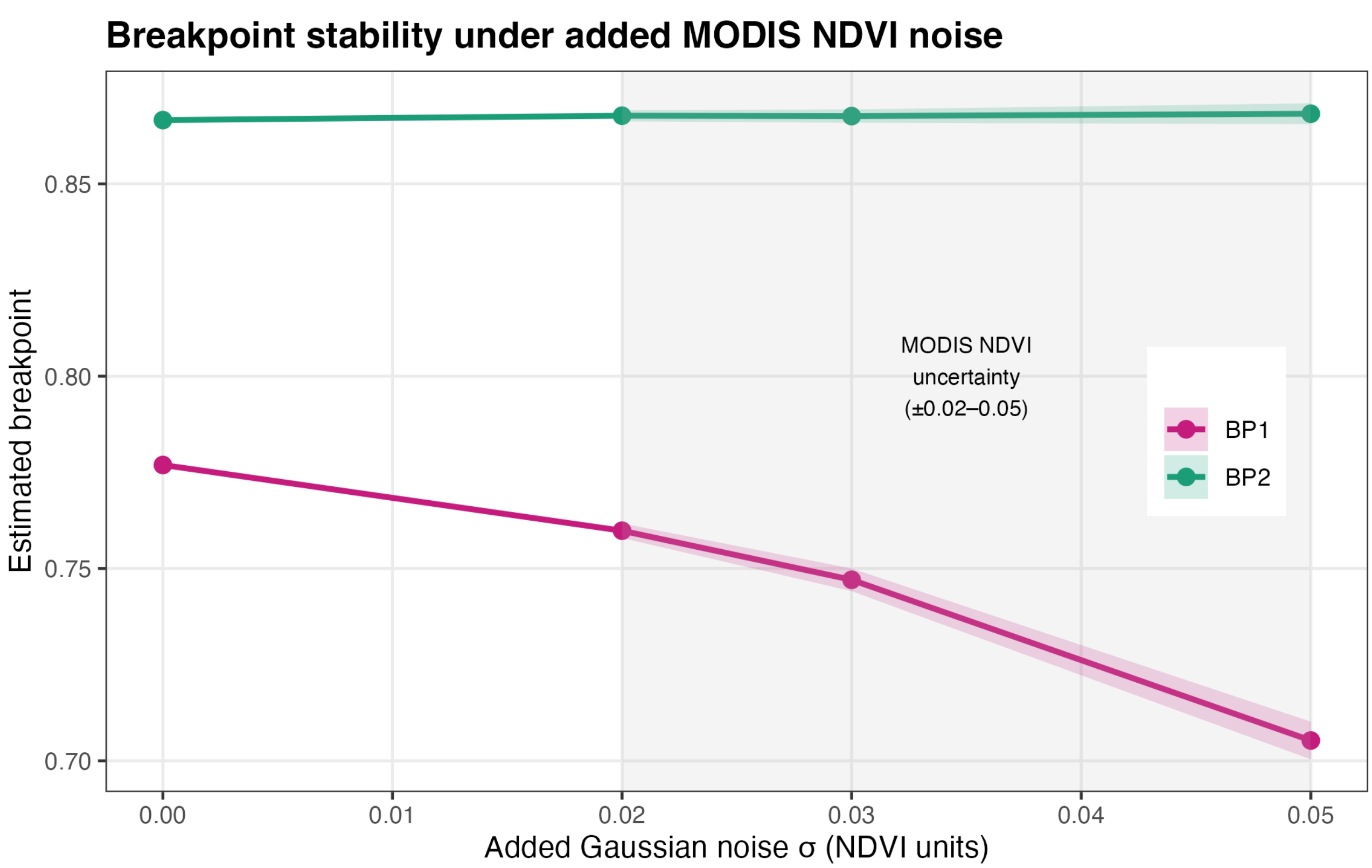

Figure S9. Monte-Carlo propagation of MODIS NDVI sensor noise through the breakpoint estimates.

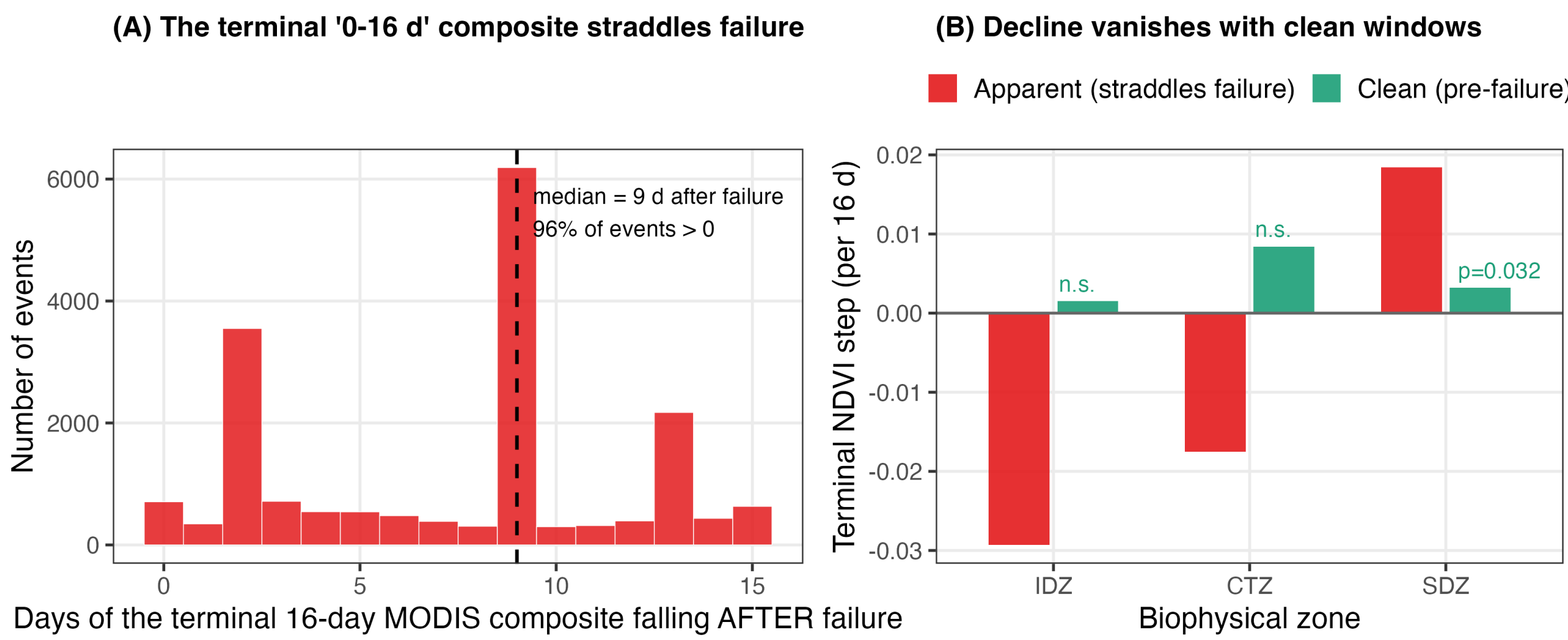

Figure S10. Post-failure compositing contamination of the terminal pre-failure NDVI window: (a) the terminal "0–16 day" 16-day composite straddles the failure date (a median of 9 days fall after failure; 96% of events); (b) the apparent zone-ordered terminal decline vanishes when the analysis is restricted to clean, fully pre-failure windows.

Supplemental Tables

Table S1. Bootstrap confidence intervals for the NDVI breakpoint estimates.

Breakpoint	Estimate	CI_Lower	CI_Upper	Boot_SE	Success_Rate
1	0.7692	0.756	0.7826	0.07178	1
2	0.8681	0.8591	0.8762	0.0381	1

Table S2. Cross-tabulation of NDVI-defined (rows) versus EVI-defined (columns) biophysical zones. Agreement is high for the IDZ, but the CTZ and SDZ boundaries fall at different index values because NDVI and EVI saturate differently at high canopy density (Figure S2).

NDVI zone ↓ / EVI zone →	CTZ	IDZ	SDZ	Total
CTZ	1,385 (33.5%)	2,740 (66.2%)	14 (0.3%)	4,139 (100.0%)
IDZ	71 (1.4%)	4,827 (98.6%)	0 (0.0%)	4,898 (100.0%)
SDZ	1,599 (46.7%)	1,723 (50.4%)	100 (2.9%)	3,422 (100.0%)
Total	3,055 (24.5%)	9,290 (74.6%)	114 (0.9%)	12,459 (100.0%)

Table S3. NDVI breakpoints across data-quality (QA) and geographic subsets (missing-data robustness).

Subset	n	BP1	BP2
QA=0 (strict)	9,168	0.7691	0.8662
QA<=1 (full reliable)	12,459	0.7692	0.8681
Excl. monsoon Asia	9,299	0.7724	0.8647
Asia/Oceania	3,811	0.7615	0.8763
Americas	4,368	0.7619	0.8685

Table S4. Breakpoint sensitivity to binning and kernel-density bandwidth choices.

Method	BP1	BP2	R2
hist_75bins	0.7699	0.8668	0.9583
hist_100bins	0.767	0.8683	0.9543
hist_125bins	0.7773	0.8663	0.9567
hist_150bins	0.7692	0.8681	0.9569
hist_175bins	0.7775	0.8661	0.9556
hist_200bins	0.779	0.8657	0.9534
KDE_SJ_x0.5	0.7741	0.8662	0.957
KDE_SJ_x1.0	0.7718	0.8668	0.9624
KDE_SJ_x2.0	0.7641	0.8673	0.9725
KDE_nrd0_x0.5	0.77	0.867	0.9649
KDE_nrd0_x1.0	0.7587	0.8675	0.9771
KDE_nrd0_x2.0	0.7226	0.8673	0.9874

Table S5. Monte-Carlo propagation of MODIS NDVI sensor noise through the breakpoints.

Sigma	BP1_mean	BP1_sd	BP2_mean	BP2_sd
0	0.7769	NA	0.8666	NA
0.02	0.7598	0.001892	0.8677	0.001459
0.03	0.7471	0.002938	0.8676	0.001723
0.05	0.7053	0.004891	0.8682	0.002707

Table S6. Test for post-failure compositing contamination of the apparent pre-failure NDVI decline: apparent (event-straddling window) versus clean-window terminal NDVI change, by zone.

Zone	n	Apparent ΔNDVI	Post-event days (med.)	Clean ΔNDVI	Clean anomaly	p (Wilcoxon)
IDZ	4,890	-0.0293	9	0.0015	0.0027	0.191
CTZ	4,143	-0.0175	9	0.0084	-0.0006	0.335
SDZ	3,426	0.0184	9	0.0032	-0.0003	0.0317

Table S7. Pairwise zone NDVI-change significance tests.

Comparison	U	p	r	Mean$_1$ (%)	Mean$_2$ (%)	n_1	n_2	$p_{\mathrm{Bonf.}}$
IDZ vs CTZ	8.48e+06	2.08e-05	0.053	-2.3	-1.65	4,501	3,981	6.24e-05
IDZ vs SDZ	5.427e+06	9.81e-104	0.284	-2.3	2.23	4,501	3,370	2.94e-103
CTZ vs SDZ	3.332e+06	1.86e-303	0.503	-1.65	2.23	3,981	3,370	5.58e-303

Table S8. Grid-based Random Forest model summary.

grid_size_deg	n_grid_cells	R2_cv	RMSE_cv	PDP_peak_NDVI	non_monotonic
0.5	364	0.446	1.088	0.86	No

Table S9. Grid-based Random Forest feature importance.

Feature	Importance
mean_EVI	0.1642
mean_elev	0.1293
mean_NDVI	0.1212
mean_EVI_change	0.1184
mean_TWI	0.1051
mean_TRI	0.0871
mean_TPI	0.083
mean_NDVI_change	0.0825
mean_slope	0.0587
mean_tree_cover	0.0505

Table S10. Grid-based Random Forest partial dependence on NDVI (PD = partial dependence; the single NDVI sequence continues across the column pairs from left to right).

NDVI	PD	NDVI	PD	NDVI	PD	NDVI	PD	NDVI	PD
0.3496	1.972	0.4537	1.981	0.5578	2.029	0.6619	1.964	0.7659	1.906
0.36	1.973	0.4641	1.985	0.5682	2.008	0.6723	1.956	0.7764	1.908
0.3704	1.973	0.4745	1.985	0.5786	2.008	0.6827	1.956	0.7868	1.91
0.3808	1.973	0.4849	1.986	0.589	2.006	0.6931	1.952	0.7972	1.925
0.3913	1.973	0.4953	1.986	0.5994	2.001	0.7035	1.95	0.8076	1.975
0.4017	1.974	0.5057	1.982	0.6098	2.001	0.7139	1.928	0.818	2.113
0.4121	1.974	0.5162	1.983	0.6202	2.004	0.7243	1.914	0.8284	2.267
0.4225	1.974	0.5266	1.985	0.6306	1.998	0.7347	1.913	0.8388	2.319
0.4329	1.973	0.537	2.012	0.641	1.989	0.7451	1.905	0.8492	2.314
0.4433	1.974	0.5474	2.029	0.6515	1.978	0.7555	1.899	0.8596	2.32

Table S11. Random Forest feature importance with terrain variables.

Feature	Importance	Is_Terrain
EVI_1	0.5005	False
NDVI_change_rate_1_to_2	0.2076	False
Hansen_Tree_Cover_2000_Percent	0.1571	False
LAI_1	0.03811	False
Elevation_m	0.02165	True
EVI_change_rate_1_to_2	0.01693	False
TRI	0.01244	True
LAI_change_rate_1_to_2	0.0107	False
TPI_500m	0.007925	True
Slope_deg	0.006948	True
Climate_Zone_Code	0.006183	False
LC_Code	0.005633	False
TWI	0.005444	True
Season_Code	0.002884	False

Table S12. Random Forest terrain comparison, including training versus cross-validated R^2 (overfitting assessment).

Model	R2_train	R2_cv	RMSE_cv	N_features	N_events	Notes
Without terrain	0.968	0.712	0.171	9	11,194	pixel-level RF; train-CV gap typical of RF
With terrain	0.968	0.717	0.17	14	11,194	delta R2 = +0.005; slope ranked 10th/14
Grid-based (0.5deg)	0.842	0.446	1.088	10	364	aggregation loss, not overfitting; n=364 cells

Table S13. Slope-stratified zone distribution analysis.

Slope class	N	IDZ n	IDZ %	CTZ n	CTZ %	SDZ n	SDZ %	Peak NDVI	In CTZ
<15°	3,170	2,298	72.5	528	16.7	344	10.9	0.591	No
15–30°	5,603	1,900	33.9	1,991	35.5	1,712	30.6	0.867	Yes
30–45°	3,388	621	18.3	1,508	44.5	1,259	37.2	0.87	No
>45°	283	58	20.5	114	40.3	111	39.2	0.87	No

Table S14. GGIG Catalog metadata summary.

Property	Value
N_events	473
Date_range_start	2017-01-16
Date_range_end	2023-10-20
Primary_country	China
NDVI_coverage_pct	37.4
Mean_NDVI	0.667
Mean_slope_deg	14.4
Mean_elevation_m	219

Table S15. Mann-Kendall trend test results for temporal NDVI changes.

Biophysical zone	N tested	Median τ	Mean τ	Prop. sig. decline	Prop. sig. increase
IDZ	4,560	-0.2	-0.09989	0.05658	0.02675
CTZ	3,976	-0.2	-0.086	0.03974	0.02968
SDZ	3,270	0.2	0.1128	0.0104	0.02599

Table S16. Moran's I spatial autocorrelation test results.

Statistic	Value	Expected	Variance	p	N	N_{eff}	k	Interpretation
Moran's I	0.6246	-0.0002	3.49e-05	0	5,000	1,155	10	Significant autocorrelation
